# Topological Scaffold‐Based Trispecific Recombinant Protein‐Drug Conjugates for Solid Tumor Eradication

**DOI:** 10.1002/advs.202501093

**Published:** 2025-08-13

**Authors:** Huiyi Jiang, Yang Yuan, Xiaoke Zhang, Weizhi Chen, Hui Mao, Baorui Liu, Xiqun Jiang

**Affiliations:** ^1^ MOE Key Laboratory of High Performance Polymer Materials & Technology and State Key Laboratory of Analytical Chemistry for Life Science and Department of Polymer Science and Engineering College of Chemistry and Chemical Engineering Nanjing University Nanjing 210093 P. R. China; ^2^ Department of Radiology and Imaging Sciences Emory University Atlanta GA 30322 USA; ^3^ The Comprehensive Cancer Centre of Drum Tower Hospital Medical School of Nanjing University Nanjing 210000 China

**Keywords:** antitumor therapy, drug delivery, nanobody, recombinant protein, trispecificity

## Abstract

Topology structure of proteins plays vital roles on their bioactivity and property. However, the understanding of how the biological effect of active targeting groups varies with different spatial positions in the recombinant protein scaffold is poor. Here a three trispecific recombinant heterotrimeric proteins and their drug conjugates are reported with a star and linear topology structure prepared by genetically encoded fusion and SpyTag‐SpyCatcher technology, which can simultaneously bind the human epidermal growth factor receptor 1 and 2 as well as integrin α_v_β_3_ on the surface of cancer cells. The biological difference of heterotrimeric proteins with different topologic structures is evaluated. Given the unique topological structure and superior biologic effects, star‐shaped trispecific recombinant proteins can specifically deliver the covalently linked payload, 7‐ethyl‐10‐hydroxycamptothecin, into HeLa tumor, leading to complete tumor eradication, while linear structural ones can only control tumor growth to some extent. The remarkable antitumor activity of the designed recombinant protein‐drug conjugate with star‐shaped structure in both small and large tumor models can be attributed not only to the triple targeting against EGFR, HER2, and integrin α_v_β_3_, but also to the “site effect” of targeting elements in heterotrimeric fusion proteins brought by the unique topological structure. The results suggest that trispecific heterotrimeric recombinant proteins with a star‐shaped topological structure is a promising drug conjugate platform.

## Graphical abstract

A novel tri‐specific recombinant protein and its drug conjugate are developed, named sRP‐EHI and sRPDC‐EHI, which feature a star‐shaped topological structure. It can simultaneously bind the EGFR, HER2, and integrin α_v_β_3_ overexpressed in cancer cells. sRPDC‐EHI effectively delivers SN38 into tumor cells, showing promising tumor accumulation and eradication potential in tumor‐bearing mice.

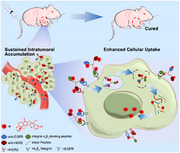

## 1 Introduction

Antibody‐drug conjugates (ADCs), which combine the specificity of antibodies with the potency of highly cytotoxic agents, are promising therapeutic agents, especially for solid tumors treatment.^[^ ^1^ ^]^ Given the highly cytotoxic payload, potentially reducing the side effects by improving the targeting specificity is tremendously important. In ADCs, conventional targeting carriers are monoclonal antibodies, which are Y‐shaped multidomain proteins with three functional components, one Fc region and two identical antigen‐binding units.^[^ ^2^, ^3^ ^]^ Despite the successful application in the treatment of various cancers, these agents have several limitations. Examples of these are suboptimal specificity of monoclonal antibodies to corresponding antigens due to the single antigen recognition and the heterogeneous antigen expression in solid tumor,^[^ ^4^, ^5^ ^]^ drug resistance associated with the signaling crosstalk between the objective targets and other targets,^[^ ^6^, ^7^, ^8^ ^]^ activation of alternate molecular pathways of cancer cells through the compensation mechanism and acquired changes in the cancer microenvironment.^[^ ^9^ ^]^ These circumstances bring great challenges for the design of targeting components in ADCs. To address these drawbacks of monoclonal antibody‐based ADCs, bispecific and trispecific antibodies, which are able to bind to more than one target, have been developed.^[^ ^10^ ^]^

Multispecific antibodies capable of simultaneous binding to multiple targets offer enhanced targeting specificity, blockade of signaling crosstalk, increased payload uptake, and reduced off‐target effects, thereby minimizing potential side effects. These attributes provide promising therapeutic opportunities to recognize and eliminate a broader spectrum of tumor cells while partially addressing the challenge of receptor expression heterogeneity.^[^ ^11^, ^12^, ^13^ ^]^More importantly, their capacity to engage multiple distinct targets simultaneously can amplify anticancer activity through synergistic effects, which cannot be achieved by drug combinations or targeting individual antigens alone. However, the larger size of bispecific and trispecific antibody scaffold may cause aggregation and bring potential immunogenicity compared to small molecules, which can be rapidly cleared. Moreover, the larger size of multi‐specific antibodies shows poor intratumoral penetration and distinct heterogeneous drug distribution in the tumor mass, as do single‐targeting ADCs.^[^ ^14^, ^15^ ^]^ Considering these challenges mentioned above in mono‐, bi‐, and tri‐specific antibody‐based ADC, small antibody formats or other targeting structure scaffolds derived from proteins may be a better selection for ADC development.

Recently, the progress in recombinant protein engineering leads to the creation of bispecific recombinant protein‐drug conjugates (RPDCs) which combine small targeting units such as single‐chain variable fragments from two different monoclonal antibodies, two single‐domain nanobodies, peptides, and chemically linked cytotoxic agents.^[^ ^16^, ^17^ ^]^ The large size and complex structure of conventional antibodies pose significant challenges for developing multispecific ADCs, particularly in controlling their topology. By virtue of improving performance and reducing molecular size, these RPDCs hold great promise to overcome the drawbacks of traditional ADCs and enhance their treatment efficiency in solid tumors.^[^ ^18^ ^]^ Moreover, currently, linear linkage or fusion of two different targeting domains is a prevalent strategy for the structure design of bispecific RPDC, which means that the targeting domains are tandemly linked by genetic engineering technology. However, for trispecific RPDCs, this straightforward linear tandem design will unavoidably result in “site effect” of targeting domains, and may make the molecular functions inefficient or biological activity weakness due to site variation of targeting domains. On the other hand, the fusion of three specific domains into a single protein molecule with non‐linear tandem structure by biotechnology is a great challenge, and rare work has addressed this issue. The understanding how the biological activity of targeting groups varies with different spatial positions is an urgent need.

**Figure 1:**
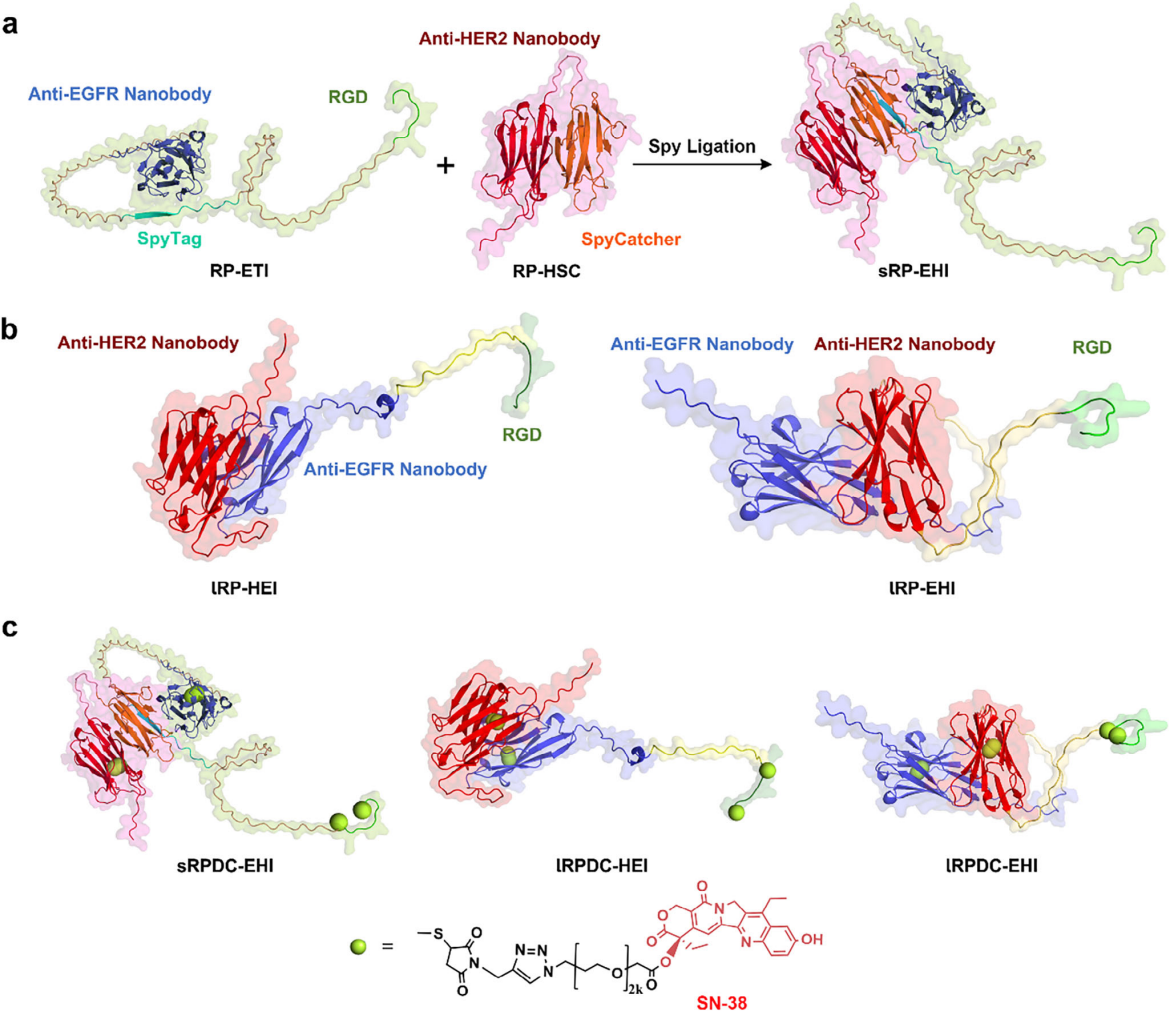
Design and preparation of RPs and RPDCs. a) Schematic representation of sRP‐EHI, a trispecific RP with a star‐shaped topological structure formed through SpyTag and SpyCatcher reaction. b) Schematic representation of two linear tri‐specific RPs, lRP‐HEI and lRP‐EHI, with different tandem structures. c) Schematic diagrams of sRPDC‐EHI, lRPDC‐EHI, and lRPDC‐HEI. The recombinant protein structures were modeled by AlphaFold 3.

Here, we report three trispecific recombinant heterotrimeric proteins (RPs), which are comprised of two single‐domain nanobodies against epidermal growth factor receptors 1 (EGFR) and 2 (HER2), respectively, and one integrin‐binding cyclic nonapeptide RGD (**Figure** **1**), can simultaneously bind the EGFR, HER2, and integrin α_v_β_3_ overexpressed in cancer cells. The crosstalk between EGFR, HER2, and integrin α_v_β_3_ makes it feasible to improve therapeutic outcomes through simultaneously blocking these three targets. Among three recombinant proteins (RPs), two have a linear structure, but the sites of each targeting domain in molecules are different. The third one has star‐ or Y‐shaped structure in which each targeting domain is located at the terminal site of the chain. Structurally, the impact of steric hindrance on each targeting domain should be minimized through a star‐shaped design, which can create more opportunities to bind targets and enter cells. Thus, the biological difference of heterotrimeric RPs and RPDCs with different topological structures can be compared. Subsequently, a cytotoxic payload, 7‐ethyl‐10‐hydroxycamptothecin (SN38), is conjugated to the RPs through a polyethylene glycol (PEG) linker to generate recombinant proteins‐drug conjugates (RPDCs). The in vitro and in vivo antitumor effect evaluation showed that star‐shaped RPDC eradicated HeLa tumors with tumor volumes of 50–100 mm^3^ and completely suppressed the growth of larger HeLa tumors with an average volume of more than 100 mm^3^. In sharp contrast, linear RPDCs only displayed a limited antitumor efficacy. The large size and complex structure of conventional antibodies pose significant challenges for developing multispecific ADCs, particularly in controlling their topology. To our knowledge, there is no multispecific ADC reported, although several dual‐specific ADCs are currently under clinical evaluation. In this manuscript, we introduce a trispecific recombinant protein‐drug conjugate (RPDC) with distinct topological architectures and demonstrate their impact on biological activities. Overall, this study not only provides a comparative insight into the impact of topological architecture on RPDC functionality but also proposes a versatile protein engineering platform for developing next‐generation trispecific RPDCs. More importantly, this platform is modular, adaptable, and can be tailored for various tumor types by altering binding domains or payloads, thus offering potential for precision oncology.

## 2 Results and Discussion

### 2.1 Preparation of Recombinant Proteins (RPs)

To prepare trispecific RPs with different topological structures, a star‐shaped trispecific heterotrimeric RP was initially designed. We first constructed two RP modules, RP‐ETI and RP‐HSC (Figure 1a; Figure S1, Supporting Information). RP‐ETI contained EGFR nanobody and RGD cyclic nonapeptide, which were at the two ends of the RP‐ETI chain, respectively, while the SpyCatcher moiety was inserted in the middle of the chain. For RP‐HSC, HER2 nanobody was linked with SpyTag moiety. The molecular weights of RP‐ETI and RP‐HSC were 26.3 and 24.3 kDa, respectively, characterized by sodium dodecyl sulfate‐polyacrylamide gel electrophoresis (SDS‐PAGE) (Figure S2a, Supporting Information). Accordingly, through the biological orthogonal reaction between SpyCatcher in RP‐ETI and SpyTag in RP‐HSC,^[^ ^19^ ^]^ a star‐ or Y‐shaped trispecific RP was generated, named sRP‐EHI (Figure 1a). SDS‐PAGE and Q‐TOF showed that the molecular weight of sRP‐EHI was 50.5 kDa (Figure S2b and S3, Supporting Information), which is consistent with its theoretical molecular weight (50.6 kDa), confirming its successful preparation. Thus, sRP‐EHI positioned all three targeting moieties (EGFR and HER2 nanobodies and RGD cyclic nonapeptide) at three chain terminal sites, respectively, displaying a feature that cannot be achieved with linear tandem structures.

In parallel, two linear trispecific heterotrimeric RPs, lRP‐HEI and lRP‐EHI, with different tandem structures, were also prepared (Figure 1b). Both lRP‐EHI and lRP‐HEI had three targeting moieties (two nanobodies and one ligand), as same as sRP‐EHI. The significant difference between lRP‐EHI and lRP‐HEI is that the EGFR‐targeting element is in the N‐terminal of lRP‐EHI, while the HER2‐targeting moiety is in the N‐terminal of lRP‐HEI. Both lRP‐EHI and lRP‐HEI were characterized by SDS‐PAGE (Figure S2c, Supporting Information) and Q‐TOF (Figures S4 and S5, Supporting Information). The molecular weights were 32.14 and 32.41 kDa, respectively. Thus, we had prepared three RPs with star‐shaped and linear topological structures and different sites of targeting elements. The 3D structures of sRP‐EHI, lRP‐HEI, and lRP‐HEI were modeled using AlphaFold 3. As shown in Figure 1, three targeting moieties (anti‐EGFR nanobody, anti‐HER2 nanobody, and RGD peptide) were oriented in different spatial direction in sRP‐EHI, which provide accessibility for each targeting domain to bind its receptor. In contrast, the middle targeting domains in linear RPs, such as anti‐EGFR nanobody in lRP‐HEI and anti‐HER2 nanobody in lRP‐EHI, showed reduced receptor accessibility due to the steric hindrance. The binding affinities of sRP‐EHI, lRP‐HEI, and lRP‐EHI for EGFR, HER2, and integrin α_v_β_3_ were evaluated using bio‐layer interferometry (BLI) for EGFR, HER2, and isothermal titration calorimetry (ITC) for integrin α_v_β_3_ (Figures S6 and S7, Supporting Information). Notably, sRP‐EHI and lRP‐EHI exhibited comparable dissociation constants (*K_D_*) for EGFR, with values of 7.77 and 4.41 nM, respectively, whereas lRP‐HEI displayed significantly weaker affinity, with a*K_D_* of 75.8 nM. For HER2, sRP‐EHI, and lRP‐HEI displayed similar *K_D_* values of 6.99 and 9.18 nM, respectively, while lRP‐EHI demonstrated reduced affinity, with a*K_D_* of 22.3 nM. In contrast, sRP‐EHI, lRP‐EHI, and lRP‐HEI exhibited comparable binding affinities for integrin α_v_β_3_, with *K_D_* values of 1.19, 9.01, and 6.33 nM, respectively. These results indicate that positioning functional domains at the terminal regions of a multispecific recombinant protein construct enhances their accessibility to target receptors, leading to stronger binding interactions. Conversely, centrally located domains may encounter steric hindrance, diminishing binding efficacy. The star‐shaped topology of sRP‐EHI, with all functional moieties situated at terminal positions, facilitates robust binding to EGFR, HER2, and integrin α_v_β_3_, highlighting the advantage of this structural design.

### 2.2 Synthesis of Recombinant Protein‐Drug Conjugates (RPDCs)

Irinotecan, a type of topoisomerase I inhibitor, has been used to manage and treat various solidtumors including colorectal cancer, breast cancer, lung cancer and pancreatic cancer.^[^ ^20^ ^]^7‐ethyl‐10‐hydroxycamptothecin (SN38),^[^ ^21^ ^]^the active metabolite of irinotecan, exhibits a more potentantitumor activity.^[^ ^22^ ^]^ However, the poor solubility and pharmacokinetic characteristics as wellas seriously side effect of SN38 severely hampered its clinical applications. Specifically targeted delivery of SN38 to tumor tissues is a promising strategy to expand its therapeutic window and consequently improve its anticancer effect in vivo.^[^ ^23^ ^]^ Therefore, SN38 was chosenas a payload for our RPDCs.

To conjugate with the RPs mentioned above, SN38 was reacted with end‐functionalized polyethylene glycol (PEG, molecular weight 2000 Da) to generate SN38‐PEG‐Mal having a maleic anhydride end, as illustrated in Figure S8 (Supporting Information). First, azide‐modified PEG was reacted with Boc‐protected SN38 (Figures S9 and S10, Supporting Information) through an esterification reaction between the carboxyl group of PEG and hydroxyl group of SN38 to link PEG with SN38 (Boc‐SN38‐PEG) (Figure S11, Supporting Information). After deprotection (Figure S12, Supporting Information), a maleimide moiety was introduced through a click reaction between SN38‐PEG and *N*‐propargyl maleimide, achieving SN38‐PEG‐Mal (Figure S13, Supporting Information). The PEG moiety improved SN38 water‐solubility, and the incorporated maleimide moiety was used for subsequent protein conjugation.

PEG modified SN38 was subsequently conjugated onto RPs through the click chemistry reaction between the maleimide in PEG modified SN38 and sulfhydryl in RPs in the presence of tris(2‐carboxyethyl) phosphine. The successful conjugation of the drug with the corresponding proteins was confirmed by SDS‐PAGE and Q‐TOF (Figure S14‐S17). All threeRPDCs, similar to SN38‐PEG, exhibited a characteristic ultraviolet absorption peak at 368 nm. The drug loading contents of sRPDC‐EHI, lRPDC‐HEI, and lRPDC‐EHI were measured by UV‐Vis spectrophotometer to be 2.09%, 1.43%, and 1.84%, respectively (Figure S18). The average ratios of drug‐to‐antibody (DAR) were 3.1, 1.29 and 1.72, respectively. The dynamic light scattering (DLS) analysis demonstrated the RPDCs were stable during the three days that incubation in PBS at room temperature, with no observed aggregation or degradation (FigureS19).

### 2.3 Enhanced Cellular Internalization of sRP‐EHI

**Figure 2:**
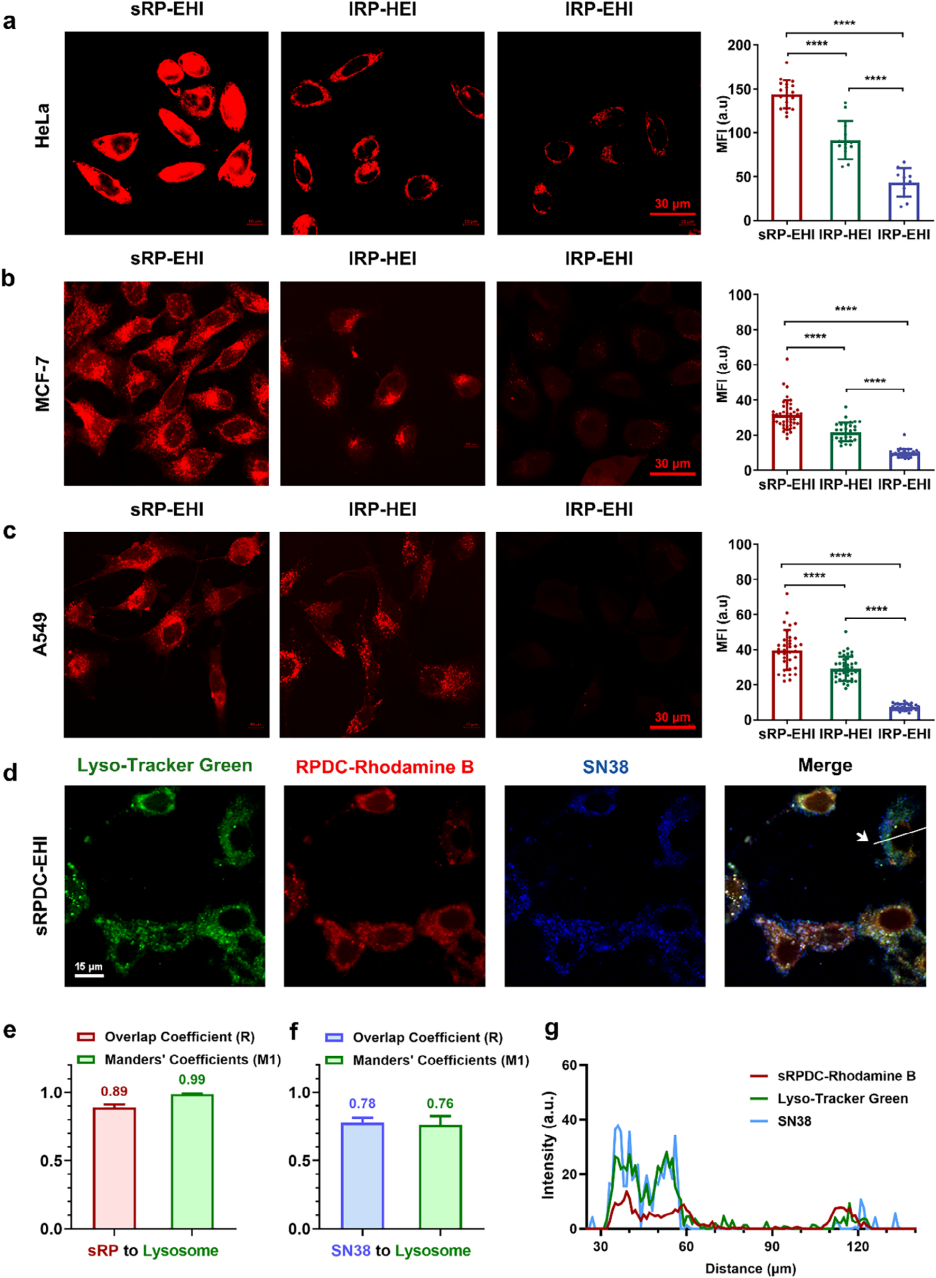
Cell internalization capacity of three RPs in three types of tumor cells. a–c) CLSM images of cellular uptake of sRP‐EHI, lRP‐HEI, and lRP‐EHI coincubating with HeLa, MCF‐7, and A549 cells for 24 h, respectively. The semi‐quantitative analysis of intracellular fluorescence intensity is shown at right. Scale bar, 30 µm. d) CLSM images of rhodamine B labeled sRPDC‐EHI (red) and Lyso‐Tracker Green (green) coincubating with HeLa for 24 h. SN38 is shown as blue. Scale bar, 15 µm. e) The overlap coefficient (R) and Manders’ coefficients between sRP and lysosome. f) The overlap coefficient (R) and Manders’ coefficients between sRP and lysosome. g) The fluorescence intensity profiles along the designated lines (g) in (d). Data are presented as mean±SD, n ≥ 12 for (a), n ≥ 30 for (b) and (c), n = 4 for (e) and (f), statistical significances were calculated using multiple *t*‐tests,^****^ *p* < 0.0001.

To evaluate the effect of topological structure on cellular internalization, star‐shaped and two linear RPs were labeled with Rhodamine B, and incubated with several types of cancer cells, including HeLa, A549, and MCF‐7 cells, which shared different expressions of EGFR, HER2, and integrin α_v_β_3_. For example, EGFR was highly expressed in HeLa and A549 cells, HER2 was overexpressed in HeLa and MCF‐7 cells, while integrin α_v_β_3_ was highly expressed in HeLa and A549 cells (Figure S20, Supporting Information). Only HeLa cells overexpressed all EGFR, HER2, and integrin α_v_β_3_. After incubation for 24 h, the cancer cells were observed using a confocal laser scanning microscope (CLSM). All the RPs could be well taken up by cancer cells, while the uptake extents were distinctly different with various cancer cells. It is worth noting that sRP‐EHI exhibited the strongest cellular uptake across all the tested tumor cell lines compared to the two linear RPs (**Figure** **2**). The semi‐quantitative analysis of intracellular mean fluorescence intensity (MFI) confirmed the superior cellular internalization capacity of sRP‐EHI over that of lRP‐HEI and lRP‐EHI. The MFI of sRP‐EHI was 1.57‐fold and 3.31‐fold of lRP‐HEI and lRP‐EHI in HeLa cells, 1.44‐fold and 3.21‐fold of lRP‐HEI and lRP‐EHI in MCF‐7 cells, and 1.37‐fold and 5.52‐fold of lRP‐HEI and lRP‐EHI in A549 cells, respectively (Figure 2a–c), suggesting the most prominent advantage of star topological structure in cellular uptake.

To further understand the uptake mechanisms of recombinant proteins (RPs), cells were pretreated with various endocytic pathway inhibitors, including sodium azide, chlorpromazine, methyl‐β‐cyclodextrin, and cytochalasin D, which are known to inhibit adenosine triphosphate‐dependent endocytosis, clathrin‐mediated endocytosis, caveolae‐mediated endocytosis, and macropinocytosis, respectively, followed with RPs incubation. As shown in Figure S21 (Supporting Information), all four tested endocytic pathways were involved in the cellular internalization of sRP‐EHI and lRP‐EHI, while only ATP‐dependent endocytosis, clathrin‐mediated endocytosis were found for lRP‐HEI.^[^ ^24^, ^25^ ^]^

Furthermore, the extent of RP uptake varied across different cancer cell lines and appeared to correlate with the distinct receptor expression levels. For instance, sRP‐EHI presented the strongest cellular uptake in HeLa cells among the three cell lines, due to overexpression of all three EGFR, HER2, and integrin α_v_β_3_ targets in HeLa cells. Thus, compared to the linear tandem structure, the star topological structure of sRP‐EHI conferred its superior spatial orientation of targeting moieties and improved receptor‐mediated cellular internalization in various cancer cells.

To investigate the intracellular trafficking behaviors of RPDCs with distinct topological architectures, we performed co‐localization studies with rhodamine B‐labeled RPDCs and lysosome tracker probe. And overlap coefficient (R) and Manders’ coefficient (M) were used for evaluating the co‐localization. As shown in Figure 2d–g and Figure S22 (Supporting Information), despite their differing topologies, the three RPDCs exhibited similar lysosome trafficking patterns within cells. The overlap coefficient (R) and a Manders’ coefficient (fraction of sRPDC‐EHI overlapping with lysosomes) of the sRPDC‐EHI to lysosomes were as high as 0.89 and 0.99 (Figure 2e). This indicates that almost all the sRPDC‐EHI was localized within lysosomes. Notably, lysosomes are rich in esterases,^[^ ^26^ ^]^ which would facilitate the cleavage of the ester linkage between the payload SN38 and RPs and thereby promote efficient drug release within the intracellular environment. To evaluate the intracellular drug release, the co‐localization of SN38 to lysosomes was also analyzed. The Manders’ coefficient for payload SN38 to lysosomes was 0.76, indicating ≈76.0% of SN38 was trafficked into lysosomes (Figure 2f). This reduced co‐localization of SN38 to lysosomes was mainly attributed to the intracellular release of SN38. Similar results were observed for lRPDC‐HEI and lRPDC‐EHI (Figure S22, Supporting Information).

### 2.4 Improved Binding of sRP‐EHI to Tumor Cell Biomarkers

**Figure 3:**
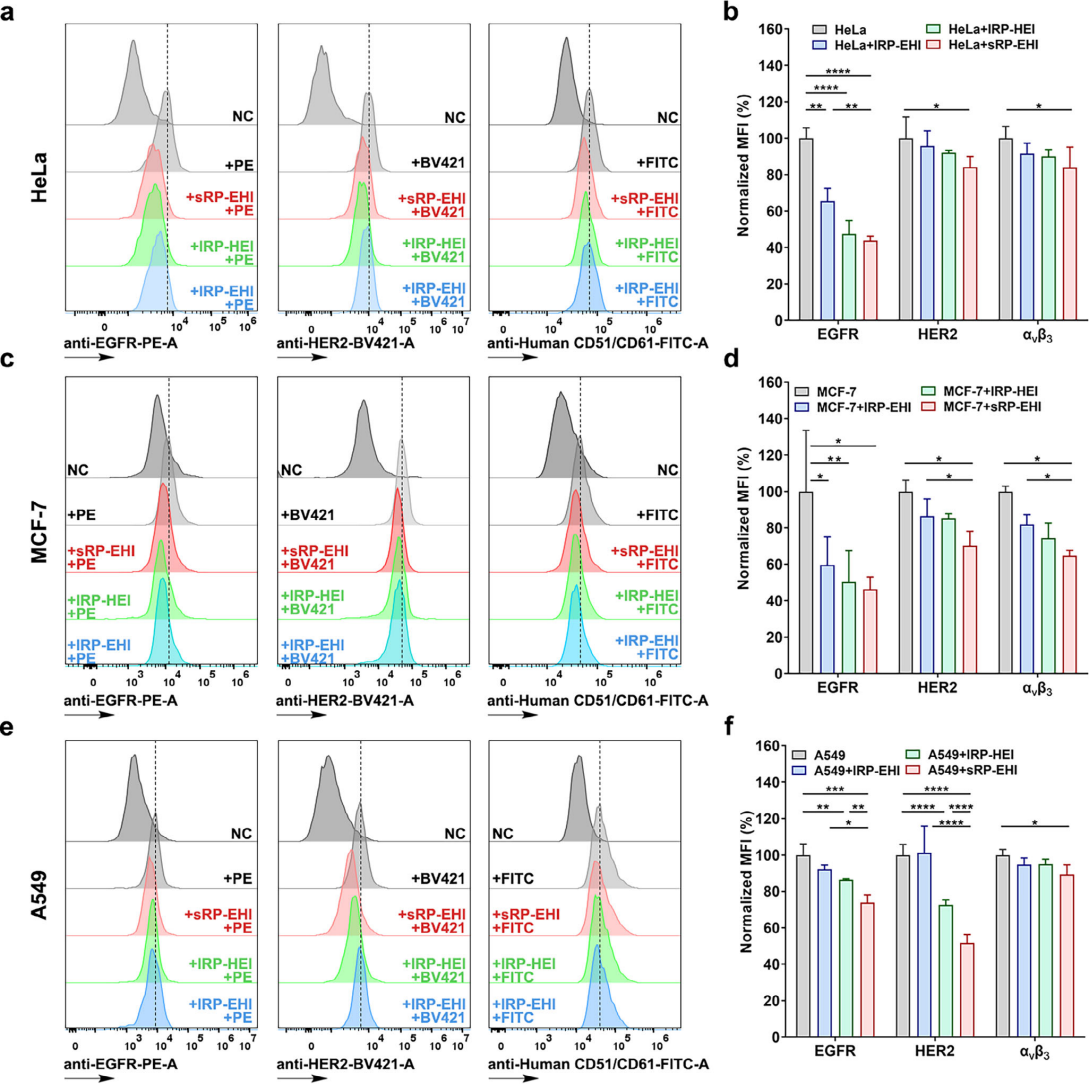
The binding capacity of three RPs to EGFR and HER2 receptors and integrin α_v_β_3_ in a,b) HeLa, c‐d) MCF‐7, and e,f) A549 cells pretreated or unpretreated with lRP‐EHI, lRP‐HEI, and sRP‐EHI by flow cytometry analysis. Data are presented as mean ± SD, statistical significances were calculated using multiple *t*‐test, n = 3, ^*^p < 0.05, ^**^p < 0.01, ^***^p < 0.001, ^****^p < 0.0001.

To validate the binding capacity of a trispecific RPs to EGFR, HER2 receptors, and integrin α_v_β_3_ in tumor cells, three RPs were co‐incubated with different cell lines (HeLa, A549, and MCF‐7) for 4 h. This allowed the biomarkers in the cells to be pre‐bound by RPs. Subsequently, anti‐EGFR‐PE, anti‐HER2‐BV421 and anti‐Human CD51/CD61‐FITC were added to specifically stain the EGFR and HER2 receptors and integrin α_v_β_3_ expressed in the cells. As shown in **Figure** **3**, the cancer cells co‐incubated with the star‐shaped sRP‐EHI in advance exhibited reduced binding of EGFR and HER2 receptors and integrin α_v_β_3_ to their corresponding antibody dyes across all cell types. This indicates that the nanobodies and ligand in the recombinant protein effectively block their corresponding targets in the cells.

Specifically, in HeLa cells (Figure 3a), the fluorescence intensity of EGFR‐bound antibodies decreased by 56%, HER2‐bound antibodies decreased by 16% and integrin α_v_β_3_‐bound decreased by 16% in the sRP‐EHI group. In MCF‐7 cells (Figure 3b), the decrease was 54% for EGFR, 30% for HER2, and 35% for integrin α_v_β_3_, while in A549 cells (Figure 3c), the decrease was 26% for EGFR, 48% for HER2, and 11% for integrin α_v_β_3_. This result demonstrates that the trispecific recombinant protein can indeed bind to EGFR and HER2 receptors as well as integrin α_v_β_3_ expressed in tumor cells, thereby achieving targeted tumor cell binding.

Notably, at equimolar concentrations, the linear recombinant proteins lRP‐EHI and lRP‐HEI also exhibited some degrees of blocking effect on the fluorescence‐labeled antibody binding to their targets. For instance, in HeLa cells (Figure 3a), the fluorescence intensity of EGFR‐bound antibodies in the lRP‐EHI group decreased by 35%, HER2‐bound antibodies decreased by 4% and integrin α_v_β_3_‐bound decreased by 8%. After co‐incubation with lRP‐HEI, the fluorescence intensity of the targeted antibodies decreased by 53%, 8%, and 10%, respectively. Obviously, their blocking efficacy was not as good as that of the star‐shaped trispecific recombinant protein, which is consistent with the cellular uptake capacities of different RPs shown in Figure 2. This difference may be related to receptor‐mediated cellular uptake pathways, once again highlighting the superior cellular uptake and receptor‐binding capabilities of the sRP‐EHI.

### 2.5 Greater Biological Activity of sRP‐EHI

**Figure 4:**
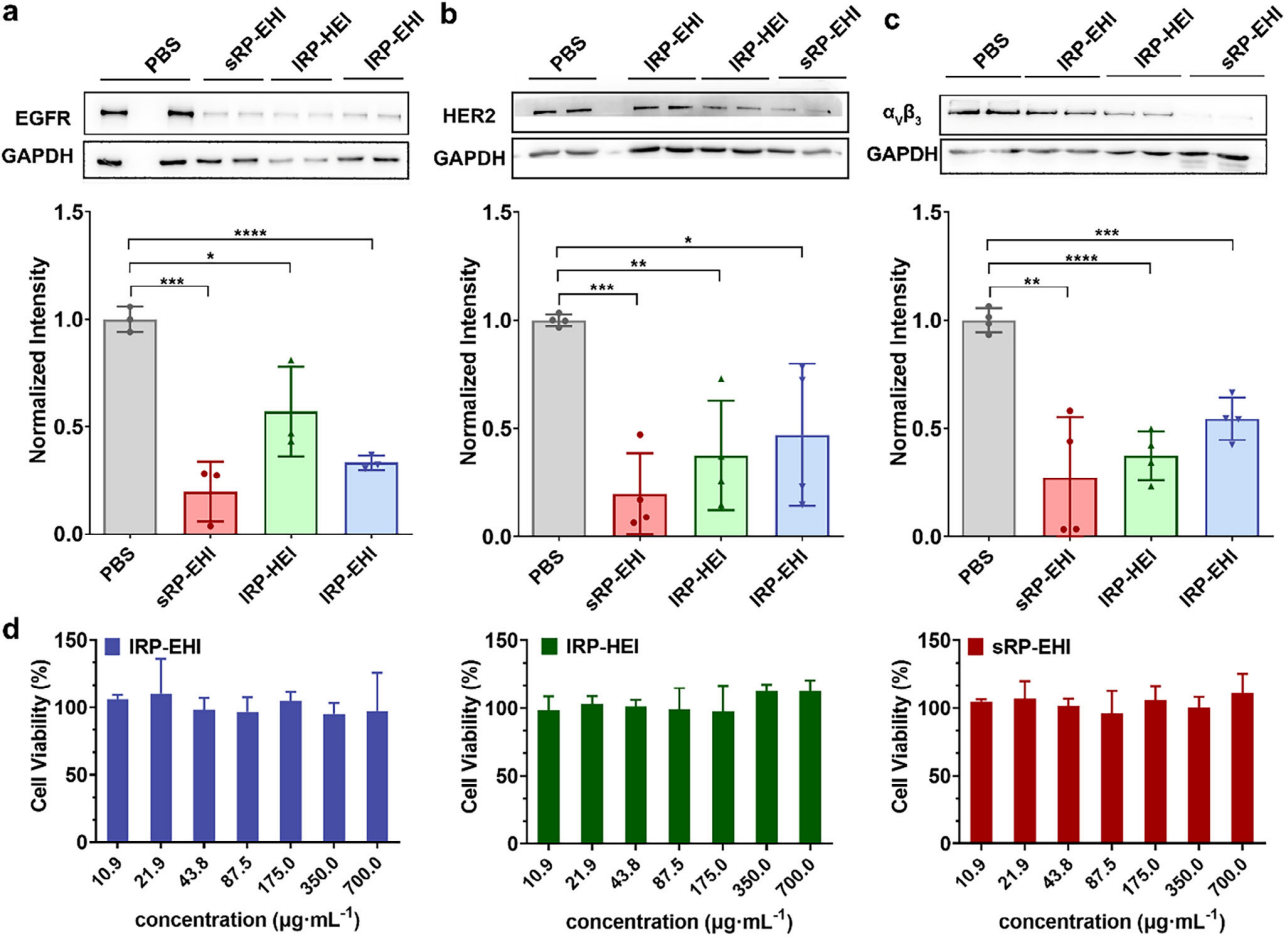
The targeted receptors expression regulation. a–c) WB analysis of EGFR, HER2, and integrin α_v_β_3_ in HeLa cells treated with different RPs for 24 h. d) In vitro cytotoxicitiy of sRP‐EHI (left), lRP‐HEI (middle), and lRP‐EHI (right) against HeLa cell determined by MTT assay after 24 h incubation. Data are presented as mean ± SD, n = 4 for (a) and (b), n = 3 for (c) and (d), statistical significances were calculated using multiple *t*‐tests, ^*^ *p* < 0.05, ^**^ *p* < 0.01, ^***^ *p* < 0.001, ^****^ *p* < 0.0001.

Next, we study the role of topological structure on the biological activity of RPs in HeLa cells since HeLa cells highly express all three targets, EGFR, HER2, and integrin α_v_β_3_ (Figure S20, Supporting Information). After respective incubation with equal amounts of different RPs for 24 h, HeLa cells were collected and lysed for protein western blotting analysis. As shown in **Figure** **4**, the trispecific RPs could simultaneously downregulate the expression level of EGFR, HER2, and integrin α_v_β_3_ in the cells. sRP‐EHI, lRP‐EHI, and lRP‐HEI downregulate the EGFR expression of HeLa cells for 80%, 67%, and 43%, respectively (Figure 4a). The HER2 expression levels of HeLa cells were downregulated by sRP‐EHI, lRP‐EHI, and lRP‐HEI for 80%, 53%, and 63%, respectively (Figure 4b). Compared linear lRP‐EHI with lRP‐HEI, lRP‐EHI with EGFR‐targeted nanobody in the N‐terminal had a better downregulation effect of EGFR than lRP‐HEI with EGFR‐targeted nanobody in the middle of the molecule. Moreover, lRP‐HEI with HER2‐targeted nanobody in the N‐terminal showed a better downregulation effect of HER2 than lRP‐EHI with HER2‐targeted nanobody in the middle of the molecule. Notably, sRP‐EHI with a star topological structure exhibited the best receptor downregulation effect for both EGFR and HER2, suggesting the importance of the terminal orientation of the targeting moiety. The integrin α_v_β_3_ expression levels of HeLa cells were downregulated by sRP‐EHI, lRP‐EHI, and lRP‐HEI for 73%, 46%, and 63%, respectively. Again, sRP‐EHI showed a higher extent of integrin α_v_β_3_ receptor downregulation than linear RPs, although all RPs had the integrin‐binding RGD peptides in the C‐terminal (Figure 4c). This may be due to the disruption of the crosstalk between EGFR, HER2, and integrin α_v_β_3_ by sRP‐EHI through downregulating all three receptors heavily, which also highlights the importance and necessity of multiple targeting several receptors by topological structure design. Additionally, no cell cytotoxicity was found for our RPs (Figure 4d). After coincubation with HeLa cells for 24 h, three RPs all showed good cytocompatibility.

### 2.6 Higher Cytotoxicity of sRPDC‐EHI

**Figure 5:**
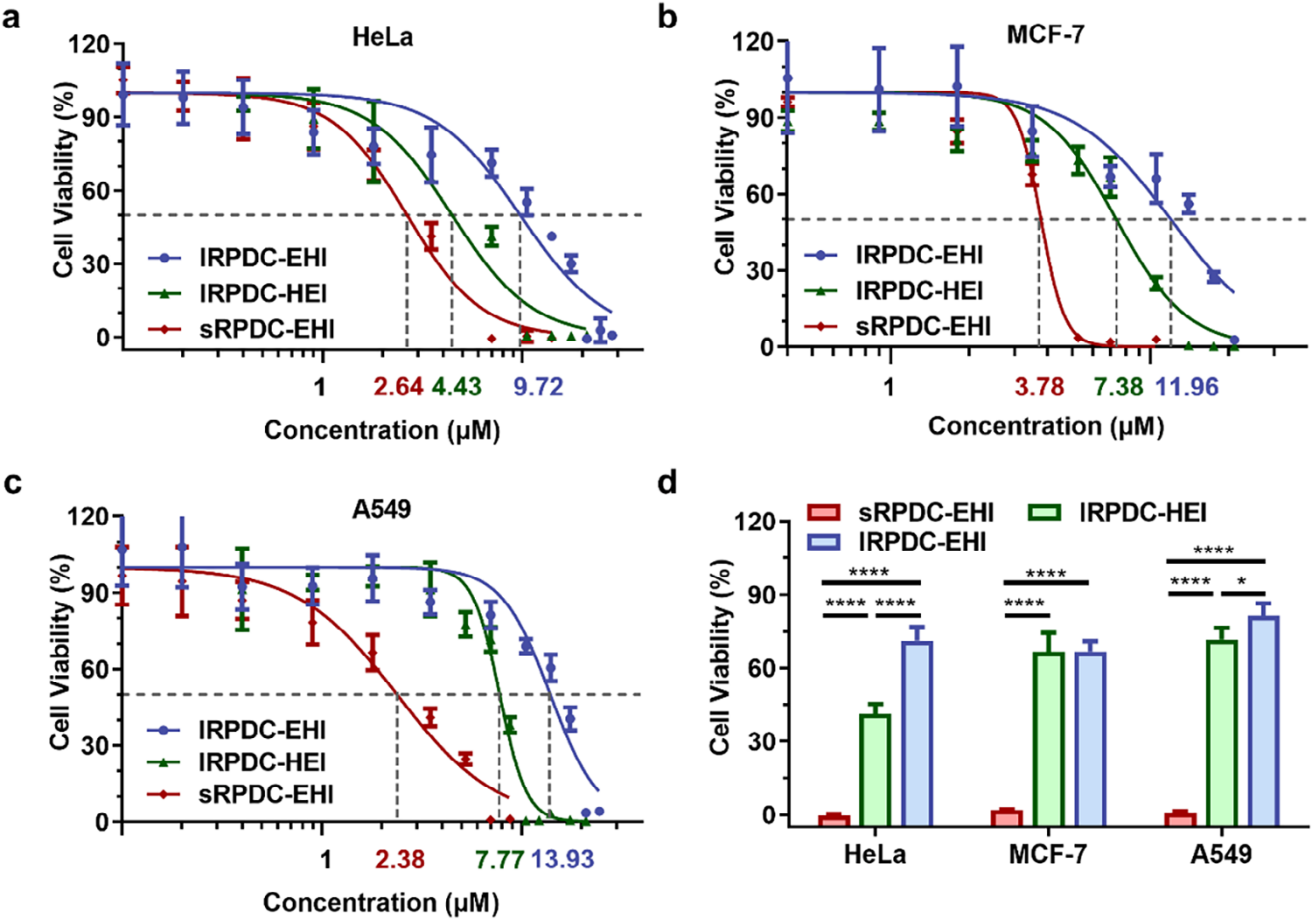
In vitro cytotoxicity of RPDCs with different topological structures. a–c) In vitro cytotoxicity and IC_50_ of lRPDC‐EHI, lRPDC‐HEI, and sRPDC‐EHI against HeLa, MCF‐7, and A549 cells after 48 h incubation. d) Cytotoxicity of lRPDC‐EHI and sRPDC‐EHI against HeLa, MCF‐7, and A549 cells at 7 µM(SN38 eq.). Data are presented as mean ± SD, n = 3, statistical significances were calculated using multiple*t*‐tests,^**^ *p* < 0.01,^***^ *p* < 0.001,^****^ *p* < 0.0001.

Before evaluating the in vivo anticancer activity of RPDCs, we assessed the in vitro cytotoxicity of the trispecific RPDCs with star‐shaped and linear structures by MTT assay. A series of concentrations of sRPDC and two lRPDCs were incubated with several cancer cells, including HeLa, MCF‐7, and A549 cells. All samples showed concentration‐dependent cytotoxicity (**Figure** **5**). After 48 h of incubation, the cytotoxicity advantage of sRPDC‐EHI compared to lRPDC‐EHI and lRPDC‐HEI in HeLa cells was more evident (Figure 5a). Similarly, sRPDC‐EHI exhibited higher cytotoxicity in MCF‐7 (Figure 5b) and A549 cells (Figure 5c) than the two linear lRPDCs. At the concentration of 7 µM (SN38 eq.), the cell viabilities of HeLa, MCF‐7, and A549 cells treated with lRPDC‐EHI were 71.27%, 66.82%, and 81.27%, respectively, while the cell viabilities for lRPDC‐HEI were 41.31%, 66.66%, and 71.68%, in sharp contrast with that for sRPDC‐EHI were 0%, 1.89%, and 0.72%, respectively (Figure 5d), suggesting the structure and biology advantage of sRPDC‐EHI. As shown in Figure 5, the half‐maximal inhibitory concentration (IC_50_) values of sRPDC‐EHI in HeLa cells for 48 h coincubation were 2.64 µM, which is 3.68‐fold lower than that of lRPDC‐EHI and 1.68‐fold lower than that of lRPDC‐HEI. Similarly, the IC_50_ values of sRPDC‐EHI in MCF‐7 and A549 cells for 48 h were 3.78 and 2.38 µM, respectively, which are 3.16 and 5.85‐fold lower than that of lRPDC‐EHI, and 3.26‐fold and 1.95‐fold lower than that of lRPDC‐HEI. These results were consistent with the results from cellular uptake and receptor expression regulation, further confirming the advantages of star‐shaped RPDCs on drug delivery.

The free drug, SN38, and its prodrug irinotecan were used as positive controls, and their cytotoxicities were also assessed by MTT assay (Figure S23, Supporting Information). After 48 h of incubation, SN38 showed significant cytotoxicity against all tested cells. The IC_50_ value for SN38 against HeLa, MCF‐7, and A549 cells were 1.55, 0.95, and 3.88 nnm, respectively (Figure S24, Supporting Information).

### 2.7 Superior Antitumor Efficiency of sRPDC‐EHI

**Figure 6:**
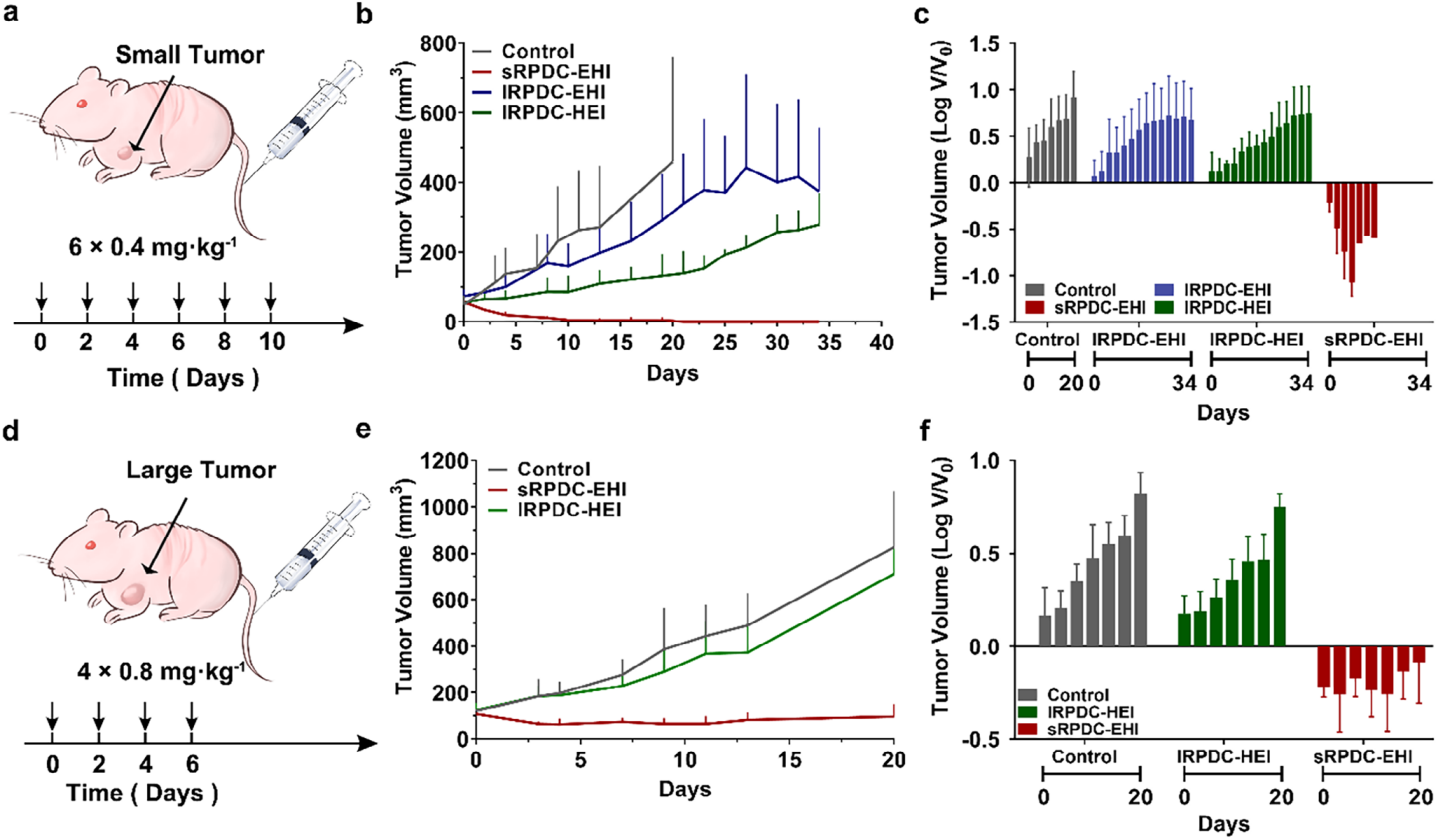
Antitumor efficacy in both small and large HeLa tumor models of three RPDCs. a) Timeline of small tumor inoculation and treatment protocol. b–c) The changes in tumor volumes from day 0 to day 34 in nude mice bearing HeLa subcutaneous tumors. d) Timeline of large tumor inoculation and treatment protocol. e,f) The changes in tumor volumes from day 0 to day 20 in nude mice bearing HeLa subcutaneous tumors. The group treated with PBS served as the control. Data are presented as mean ± SD. n = 3.

The antitumor efficiency of three RPDCs with different topological structures was investigated in a mouse model of subcutaneous xenograft HeLa tumor. As depicted in **Figure** **6**a, when the initial tumor volumes reached in the range of 50–100 mm^3^, star‐shaped RPDC, sRPDC‐EHI, and the two linear RPDCs, lRPDC‐EHI and lRPDC‐HEI, were intravenously injected into the HeLa tumor‐bearing mice at a dosage equivalent to 0.4 mg kg^−1^ of SN38. All the RPDCs were administered every two days, and a total of six injections were given. In contrast to the fast‐growing tumors in the PBS group, the tumors treated with lRPDC‐EHI were inhibited to some extent degree (Figure 6b). Differently, lRPDC‐HEI exhibited a better effect of tumor control growth than lRPDC‐EHI, suggesting the tandem site of targeting moiety in RPDC has an important effect on its biological and antitumor activity. However, neither lRPDC‐EHI nor lRPDC‐HEI could reduce the sizes of established HeLa tumors. In a sharp contrast, the tumor volumes in the sRPDC‐EHI group were reduced immediately after the first injection and continued to decrease until the tumors were disappeared (Figure 6b,c). At day 20 after the first injection, the tumor volumes for control, lRPDC‐EHI, and lRPDC‐HEI‐treated mice were 460, 338, and 140 mm^3^, while sRPDC‐EHI‐treated mice showed complete tumor eradication in the test group. The tumor inhibition rates of sRPDC‐EHI, lRPDC‐EHI, and lRPDC‐HEI were 100%, 52%, and 73%, respectively, at day 20. The excellent tumor eradication effect of sRPDC‐EHI confirms again the importance of the star topological structure on the biological activities of targeting therapeutics. In addition, no obvious change in body weight was found in the treated mice (Figure S25, Supporting Information). To further assess the biosafety and immunogenicity of RPDCs, we analyzed complete blood count (CBC, Figure S26, Supporting Information), blood biochemical parameters (Figure S27, Supporting Information), and inflammatory cytokine levels (Figure S28, Supporting Information) at day 7 following the final injection. No significant differences were observed compared to PBS control group. Additionally, histological and ultrastructural analyses of major organs revealed no evidence of injury (Figure S29, Supporting Information). These findings demonstrate the excellent biosafety profile of RPDCs treatment.

To evaluate the antitumor activity for larger tumor, we established a subcutaneous tumor model with tumor volumes exceeding 100 mm³ (Figure 6d). The mice bearing large HeLa tumors were intravenously administered with sRPDC‐EHI and lRPDC‐HEI every two days at a dosage equivalent to 0.8mg kg^−1^ of SN38 and a total of four injections were given. In sharp contrast to small tumors, large tumors share a more complicated tumor microenvironment and are usually more resistant to antitumor drugs.^[^ ^27^ ^]^ Different from the markedly antitumor effect against small tumors, lRPDC‐HEI exhibited negligible antitumor activity against large tumors. However, sRPDC‐EHI still demonstrated encouraging and persistent tumor inhibition (Figure 6 e). During the observed 20 days, a remarkable reduction of tumor volumes in the mice treated with sRPDC‐EHI was observed (Figure 6 f), and the average tumor volume reduced from 120 mm³ to less than 100 mm³ after treatment. Different from the slow‐down tumor growth behavior of other antitumor drugs and formulations,^[^ ^28^, ^29^ ^]^ our sRPDC‐EHI showed a significant shrinkage of tumor volume for a large tumor model with tumor volumes more than 100 mm³. The tumor inhibition rate of sRPDC‐EHI reached 87%, while that of lRPDC‐HEI was only 17% at day 20 after first injection for large tumors. In addition, no obvious weight change of mice wasobserved for all treatment groups at a dosage of 0.8 mg kg^−1^ SN38 equ., suggesting the dosage selection is tolerable (Figure S30).

The remarkable anti‐cancer capabilities of sRPDC‐EHI with star‐like structure in both small and large tumor models can be attributed not only to the triple targeting against EGFR, HER2, and integrin α_v_β_3_ but also to the ‘site effect’ of targeting elements brought by the unique topological structure.

### 2.8 Sustained Tumor Accumulation of sRPDC‐EHI

**Figure 7:**
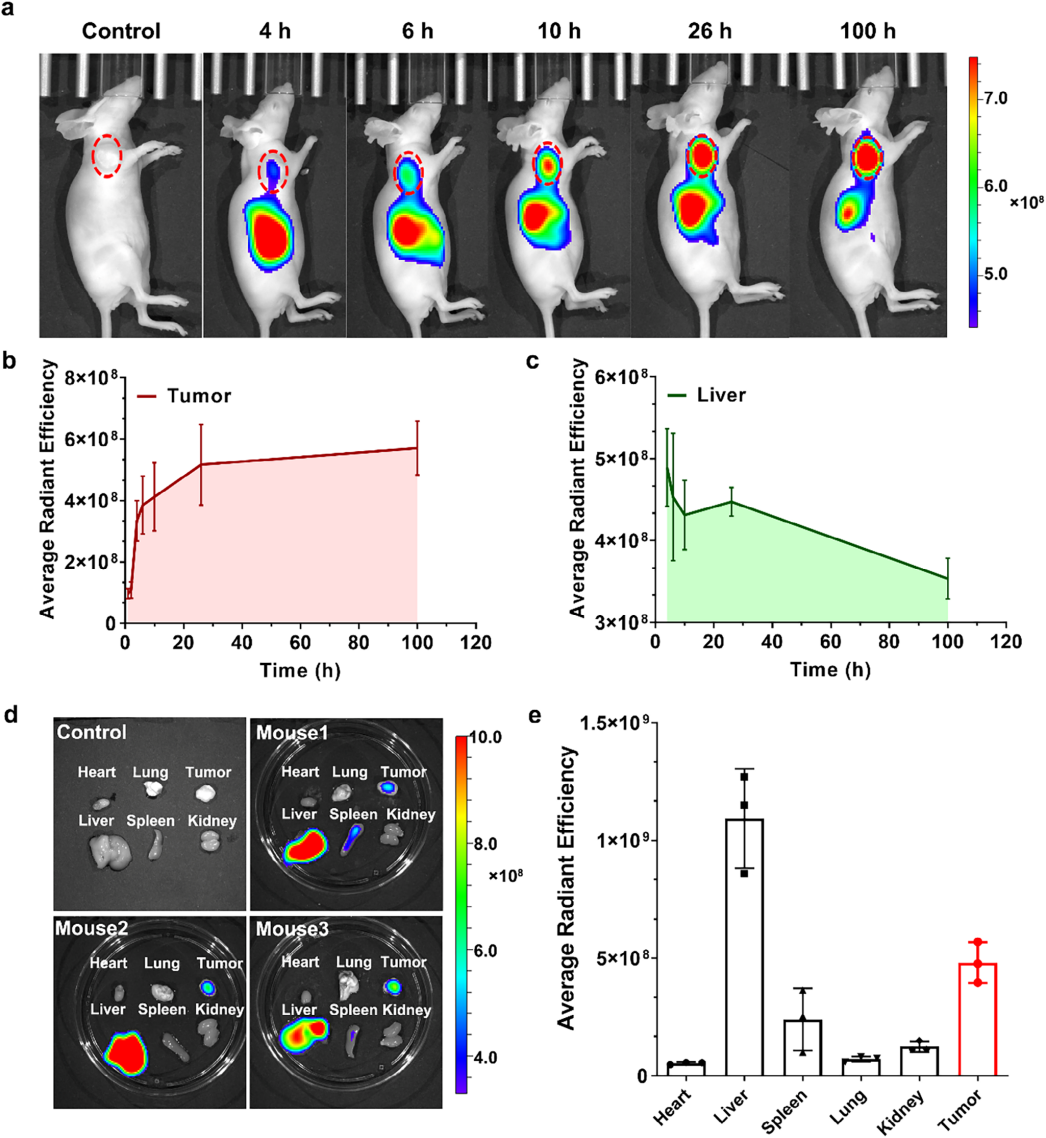
sRPDC‐EHI could continuously accumulate in tumors. a) The in vivo NIR images of HeLa tumor‐bearing mice following intravenous injection of PBS and sRPDC‐EHI‐NIR‐797 at different time points. The tumors were surrounded by red dotted lines. b,c) The average radiant efficiency in tumors and livers for sRPDC‐EHI‐NIR‐797 at different times. d) Ex vivo NIR imaging of tumors and major organs at 100 h post‐injection. e) The average radiant efficiency in tumors and major organs. Data are presented as mean ± SD, n=3.

To further analyze the mechanism behind the greater antitumor efficacy of sRPDC‐EHI, we next evaluate tumor accumulation and biodistribution of sRPDC‐EHI. To enable real‐time visualization of sRPDC‐EHI in vivo, sRPDC‐EHI labeled with near‐infrared (NIR) dye, NIR797 was administered into the HeLa tumor‐bearing mice via the tail vein. At different time points post‐injection, the tumor‐bearing mice were imaged by an NIR imaging system. As shown in **Figure** **7**a, sRPDC‐EHI was mainly accumulated in tumors and livers. sRPDC‐EHI could reach the tumor tissue at 4 h post‐injection. The intratumor fluorescence intensity increased with time, suggesting a continuous accumulation of sRPDC‐EHI. In contrast, the fluorescence intensity in the livers was time‐dependently decreased. The semi‐quantitative analysis of the average fluorescence intensity in tumors and livers further confirmed this phenomenon. The accumulation of sRPDC‐EHI in tumor tissues continuously increased as long as 100 h after a single injection which is significantly different from the in vivo behavior of traditional nanodrugs and small molecular drugs.^[^ ^30^ ^]^ The average intratumor fluorescence intensity at 100 h was ≈1.7‐fold of that at 4 h post‐injection (Figure 7b), suggesting the long circulation feature of sRPDC‐EHI. While the average fluorescence intensity in livers at 100 h was approximately 0.7 times compared of those at 4 h post‐injection (Figure 7c), indicating sRPDC‐EHI is gradually metabolized and eliminated from the body. Given the penetration depth limitation of fluorescence imaging in vivo, the tumors and major organs were collected for ex vivo NIR imaging at 100 h post‐injection (Figure 7d). Similar to the data from in vivo NIR imaging, ex vivo NIR imaging displayed that sRPDC‐EHI was mainly accumulated in livers, tumors, and spleens. The average intratumor fluorescence intensity was 9.03‐fold, 6.73‐fold, 3.87‐fold, and twofold higher than that observed in the heart, lung, kidney, and spleen (Figure 7e). The considerable intratumor drug accumulation determined the excellent tumor targeting and antitumor effect of sRPDC‐EHI.

To further explore the potential mechanism underlying intratumor drug accumulation, we conducted an in vivo pharmacokinetic analysis using Cy5.5‐labeled sRPDC‐EHI. By measuring the fluorescence intensity of mouse serum at various time points post‐administration, the in vivo half‐life time of sRPDC‐EHI was determined as 31.65 h (non‐compartmental analysis). As shown in Figure S31 (Supporting Information), the blood clearance of sRPDC‐EHI was relatively slow, particularly after 1 h, which may be one of the contributing factors to its enhanced intratumor drug accumulation. Additionally, the multiple targeting ability would further improve the intratumor drug retention.

## 3 Conclusion

In this study, we developed three trispecific recombinant proteins RPs and RPDCs targeting EGFR, HER2, and integrin α_v_β_3_, each with distinct topological architectures, and assessed their biological activities both in vitro and in vivo. Compared to the linear lRP‐EHI and lRP‐HEI, sRP‐EHI, characterized by a star‐shaped topology, exhibited significantly enhanced receptor binding and blocking effects due to reduced steric hindrance, despite all constructs sharing identical targeting moieties. A cytotoxic drug, SN38 was conjugated onto three RPs through click chemistry using a PEG as spacer, generating three recombinant protein‐drug conjugates (RPDCs). sRPDC‐EHI with a star‐like topological structure guaranteed that all three targeting moieties were at terminals and showed a significantly enhanced specific binding, cellular internalization capacity, and sustained drug accumulation in tumors, resulting a superior antitumor effect. In contrast with the growing tumors in the control, lRPDC‐HEI, and lRPDC‐EHI groups, HeLa tumors with a volume less than 100 mm^3^ were entirely eradicated after treatment with sRPDC‐EHI. More importantly, sRPDC‐EHI significantly reduced the volume of large HeLa tumors with an average volume of more than 100 mm^3^, which were resistant to lRPDC‐HEI. These results indicate the importance of topological structure in managing the biological activities of multiple specific recombinant proteins and recombinant protein‐drug conjugates. The star‐shaped RPDC constructed in this study provides a great platform for constructing trispecific drug conjugates with optimized antitumor effects.

While sRPDC effectively eradicates small tumors, it primarily inhibits the growth of larger tumors, indicating the emergence of drug resistance. Therefore, future strategies could consider developing dual‐payload RPDCs and combining them with other cancer therapeutics, such as immunotherapy, to enhance multidimensional cancer cell killing.

## 4 Experimental Section

All experimental details including statistical analysis are shown in the Supporting Information. All animal experiments were approved by the Animal Care Committee at Nanjing University (approval number: IACUC‐2003099). All procedures involving animals were conducted in strict accordance with the ethical guidelines and regulations established by this committee to ensure humane treatment and welfare throughout the study.

### Statistical Analysis—Preparations of recombinant proteins

The protein concentrations were calculated using the protein molar extinction coefficient, and the absorbance at 280 nm was measured through a UV–vis spectrum. Protein structural simulations were performed using AlphaFold3 and analyzed with PyMOL.

### Statistical Analysis—Preparation of SN38‐PEG‐Mal

The ^1^H NMR spectrum and HPLC data were analyzed using MestReNova and Origin. The ^1^H NMR spectrum was subjected to integration analysis. HPLC data were normalized based on the peak with the highest intensity.

### Statistical Analysis—Cell Internalization and Colocalization Study

Fluorescence images were analyzed by ImageJ. Data are presented as mean ± SD, n ≥ 3, statistical significances were calculated using multiple *t*‐tests, ^**^ *p* < 0.01, ^***^ *p* < 0.001, ^****^ *p* < 0.0001.

### Statistical Analysis—Flow Cytometry

Flow cytometry data were analyzed using FlowJo and GraphPad Prism. Normalized values were indicated on the respective graphs. Data are presented as mean ± SD, statistical significances were calculated using multiple *t*‐test, n = 3, ^*^p < 0.05, ^**^p < 0.01, ^***^p < 0.001, ^****^p < 0.0001.

### Statistical Analysis—Receptor‐Binding Affinity Assay

The affinities of RPs binding to EGFR and HER2 were determined through biolayer interferometry (BLI) assay using the Octet BLI system. The sample‐sensorgrams were corrected by subtracting the reference curve. Global 1:1 fitting of association‐ and dissociation curves with the analysis software revealed *K_D_*. The affinities of RPs binding to integrin α_v_β_3_ were measured through isothermal titration calorimetry (ITC) assay using a Malvern MicroCal ITC200‐09‐11‐547 instrument at 25 °C. The data recorded was analyzed using the accompanying Malvern analysis software and fit with a one‐site binding model.

### Statistical Analysis—Cell Viability, Western Blotting, and In Vivo Anti‐Tumor Experiments

All data were analyzed using GraphPad Prism. Normalized values were indicated on the respective graphs. Data were presented as mean ± SD, n ≥ 3, statistical significances were calculated using multiple *t*‐tests, ^*^p < 0.05, ^**^ *p* < 0.01, ^***^ *p* < 0.001, ^****^ *p* < 0.0001.

## Conflict of Interest

The authors declare no conflict of interest.

## Author Contributions

H.Y.J. conceived and designed the study, performed the experiments, conducted data analysis, and drafted the manuscript. Y.Y. and X.K.Z. assisted with data collection and analysis. W.Z.C. and X.Q.J. contributed to experimental design, data interpretation, and manuscript revision. H.M. and B.R.L. provided expert guidance during the revision and contributed to manuscript editing and language refinement. X.Q. J. supervised the whole project.

## Supporting information



Supporting Information

## Data Availability

The data that support the findings of this study are available in the supplementary material of this article.
