# Supplementary material for: Topological Scaffold‐Based Trispecific Recombinant Protein‐Drug Conjugates for Solid Tumor Eradication
Source: Adv Sci (Weinh). 2025 Aug 13;12(37):e01093. doi: 10.1002/advs.202501093 (PMC12499393; doi:10.1002/advs.202501093)
Supplement: Supplementary file 1 — Supporting Information [file ADVS-12-e01093-s001.pdf]

ADVANCED SCIENCE Open Access

Supporting Information

for *Adv. Sci.*, DOI 10.1002/advs.202501093

Topological Scaffold-Based Trispecific Recombinant Protein-Drug Conjugates for Solid Tumor Eradication

Huiyi Jiang, Yang Yuan, Xiaoke Zhang, Weizhi Chen, Hui Mao, Baorui Liu and Xiqun Jiang**

Supporting Information

Topological Scaffold-Based Trispecific Recombinant Protein–Drug Conjugates for Solid Tumor Eradication

Huiyi Jiang, Yang Yuan, Xiaoke Zhang, Weizhi Chen, Hui Mao, Baorui Liu, Xiqun Jiang**

H. Jiang, Dr. Y. Yang, Dr. X. Zhang, Prof. W. Chen, Prof. X. Jiang

MOE Key Laboratory of High Performance Polymer Materials and Technology, and Department of Polymer Science & Engineering, College of Chemistry and Chemical Engineering, Nanjing University, Nanjing 210093, P. R. China

E-mail: jiangx@nju.edu.cn; chenwz@nju.edu.cn

Prof. H. Mao

Department of Radiology and Imaging Sciences

Emory University

Atlanta, GA 30322, USA

Prof. B. Liu

The Comprehensive Cancer Centre of Drum Tower Hospital, Medical School of Nanjing

University, Nanjing, China.

Materials and Methods

Materials

N-propargyl maleimide, acetic acid, ethanol, trifluoroacetic acid, and esterase were purchased from Beijing InnoChem Science & Technology Co., Ltd. Dicyclohexylcarbodiimide was obtained from Shanghai Bide Pharmatech Ltd. Anti-Integrin alpha V beta 3 antibody [BV3] was procured from Jiangsu KeyGEN Biotechnology Co., Ltd. Other chemical reagents were sourced from Nanjing WANQING Chemical Glass ware & Instrument Co., Ltd. The pET-28a plasmid containing RP-ETI and RP-HSC gene sequences was prepared and supplied by Nanjing GenScript Biotech Corporation. Rabbit anti-human EGFR monoclonal antibody was purchased from ABChem. Rabbit anti-human GAPDH polyclonal antibody was obtained from Beijing InnoChem Science & Technology Co., Ltd. Fluorescent dyes such as fluorescein isothiocyanate (FITC) and NIR-797 isothiocyanate were procured from Sigma-Aldrich.

Human non-small cell lung cancer A549 cells and human cervical cancer HeLa cells were acquired from Jiangsu KeyGEN Biotechnology Co., Ltd. Human breast cancer MCF-7 cells was obtained from Wuhan Procell Life Science & Technology Co.,Ltd. Cell cultures were maintained in DMEM (Hyclone) medium supplemented with 10% fetal bovine serum (Vicent BioTech), 100 U/mL penicillin, and 100 μg/mL streptomycin (Sigma Aldrich). Female BALB/C nude mice were purchased from Nanjing Zhonghua Biotechnology Co., Ltd., and housed in a specific pathogen-free (SPF) environment. The mice were provided with water and food ad libitum under professional and pathogen-free conditions. All animal experiments were conducted in accordance with the guidelines set by the Animal Ethics and Welfare Committee of Nanjing University.

Experimental Methods

Preparations of recombinant proteins

The plasmid construction of the recombinant protein genes of lRP-HEI, RP-ETI, and

RP-HSC was completed by Shanghai GENEray (Shanghai, China). Recombinant plasmid with lRP-EHI gene was synthesized from the plasmid anti-EGFR-PET28a via polymerase chain reaction (PCR) amplification with the primers 1-2 and the plasmid anti-HER2-RGD-PET28a with the primers 3-4 as follows:

primer 1:

5' CCGCTCGAGATGCATCACCATCACCATCACCA 3'

primer 2:

5' TCATGCCATGGGTTCAGATCTTCTTCGCTGATATCAGCGAAGAAGATCTGAACCCATGGCATG 3'

primer 3:

5' CCGCTCGAGGGTATATCTCCTTCTTAAAGTTAACTTTAAGAAGGAGATATACCCTCGAGCGG 3'

primer 4:

5' CATGCCATGGGGCCAGGTGAAGCTGGTGGAGT 3'

The gene sequences of recombinant plasmids were confirmed through sequencing performed by SPRINGEN Biotechnology (Nanjing, China). The encoded protein sequences were annotated as follows: SpyCatcher (UniParc: UPI000902C26B), SpyTag (UniParc: UPI0003C63E7B), anti-EGFR nanobody (QVKLEESGGGLVQAGDSLRVSCAASGRDFSDYVMGWFRQAPGKEREFVAAISRNGLTTRYADSVKGRFTISRDNDKNMVYLQMNSLKPEDTAVYYCAVNSAGTYVSPRSREYDYWGQGTQVTVSSGSEQKLISEEDLN), anti-HER2 nanobody (GQVKLVESGGGLVQPGGSLRLSCAASGSGFSPNVMGWYRQTPGNRREWVAAANKYGTTTYADSVKGRFAISRDNAKTTVYLQMNSLKPEDTAVYYCAASTATNWDYHYWGQGTQVTVSS) and RGD (CRGDKGPDC).

All the recombinant proteins were expressed from the Echerichia coil, which were cultured the strains in Luria-Bertani (LB) medium containing kanamycin (30 μg/mL) at 37°C in a shaking incubator at 200 rpm overnight. Then the bacteria were transfered to a bigger shake flask with 400 mL TB medium and incubate for another 3 hours. Then IPTG with a final concentration of 0.5 M was added into the culture medium for

induction of proteins expression when the optical density at 600 nm (OD_{600}) reaches about 0.8. After incubation at 18°C for 24 hours, E. coli cells were harvested by centrifugation at 4°C and 8500 rpm for 10 minutes. Resuspend the cells in cold PBS buffer (pH 7.5) and sonicate on ice to lyse the cells, then centrifuge at 4°C and 13,400× g for 30 minutes to remove the insoluble cell debris.

The RPs were purified using affinity chromatography through the inserted His tag and the nickel chelate column. Crude samples were loaded in loading buffer (20 mM Tris, pH 7.5, 20 mM imidazole), the non-specific absorption was washed with washing buffer (20 mM Tris, pH 7.5, 40 mM imidazole) and the elution buffer (20 mM Tris, pH 7.5, 300 mM imidazole) was used to elute the product proteins. SDS-PAGE was used for analyzing the purity and molecular weight of each collection. The imidazole was removed by dialysis.

sRP was constructed from RP-ETI, and RP-HSC through the SpyCatcher and SpyTag mediated biological orthogonal reaction. Specifically, RP-ETI and RP-HSC in PBS with the same molar concentrations were mixed and reacted at 4 ºC overnight. The reaction was monitored by SDS-PAGE.

The purified recombinant proteins lRP-HEI, lRP-EHI, and sRP-EHI were separately stored in PBS (pH = 7.4) containing 10 mM EDTA and stored at -80ºC for further use. The protein concentrations were calculated using the protein molar extinction coefficient and the absorbance at 280 nm were measured through a UV-Vis spectrum.

Preparation of SN38-PEG-Mal

SN38-PEG-Mal was prepared through four steps as show in Figure S8.

Synthesis of Boc-SN38

1 g of hydroxycamptothecin was dissolved in 100 mL of anhydrous dichloromethane. 1 g of di-tert-butyl dicarbonate, and 5mL of anhydrous pyridine was then added, followed by stirring for 3 hours. The resulting product was washed three times with 0.5N dilute hydrochloric acid and a saturated solution of sodium bicarbonate. The

product was then extracted with dichloromethane, dried with a small amount of anhydrous sodium sulfate powder, filtered, and the solvent was removed by rotary evaporation to obtain yellow powder, Boc-SN38, which was confirmed by ^{1}H NMR spectrum (Figure S9)[1,2] and ESI MS (Figure S10), MS (Boc-SN38 H^+) = 494.20 Da, MS (Boc-SN38 H^+·DMSO) = 572.25 Da. Boc-SN38 ^{1}H NMR (400 MHz, DMSO-d_6) δ 8.22 (d, *J* = 9.2 Hz, 1H), 8.15 – 8.00 (m, 1H), 7.75 (dd, *J* = 9.2, 2.6 Hz, 1H), 7.43 – 7.19 (m, 1H), 6.53 (s, 1H), 5.45 (s, 2H), 5.35 (s, 2H), 4.09 (q, *J* = 5.3 Hz, 1H), 3.23 – 3.17 (m, 2H), 2.00 – 1.73 (m, 2H), 1.55 (s, 9H), 1.38 – 1.21 (m, 3H), 0.89 (t, *J* = 7.3 Hz, 3H).

Synthesis of the Boc-SN38-PEG

80 mg of Boc-SN38 and 267 mg of carboxylated polyethylene glycol with an azide group (PS2-CMAZ-2K, Ponsure) were dissolved in anhydrous dichloromethane, 48 µL of N, N'-diisopropylcarbodiimide and 30 mg of 4-dimethylaminopyridine were then added. The reaction mixture was stirred at room temperature for more than 12 hours. The solvent was removed by rotary evaporation, and the product was purified by size exclusion chromatography to obtain a transparent glassy solid, Boc-SN38-PEG.

Synthesis of the SN38-PEG

Next, the obtained Boc-SN38-PEG was dissolved in 1 mL of trifluoroacetic acid, and 1mL of dichloromethane was added as a co-solvent. The reaction was stirred at room temperature for more than 4 hours. The solvent was then removed by rotary evaporation, and the pH was adjusted to neutral before drying again by rotary evaporation to obtain SN38-PEG, which was confirmed by thin layer chromatography, MALDI-TOF (Figure S11) as well as HPLC (Figure S12). A C_{18} chromatographic column (4.6 mm × 250 mm, 5 µm) was used and the mobile phases were methanol (A) and acetonitrile with a 0.1% trifluoroacetic acid solution (B). A gradient elution approach was employed, the elution program commenced with 0-20 minutes (0% A~100 % A), succeeded by 20-30 minutes (100% A). The flow rate was maintained at 1.0 mL/min, with an injection volume of 10 µL Detection occurred at a wavelength of 365 nm, and the column temperature was

sustained at room temperature.

Synthesis of the SN38 -PEG-Mal

80 mg of PEG-SN38 and 4.32 mg of propargyl maleimide were dissolved in 5 mL of N, N-dimethylformamide. Subsequently, 1.1 mg of cuprous iodide and 4.7 mg of pentamethyldiethylenetriamine were added to the solution, which was then transferred to a Schlenk flask. After three cycles of vacuum and nitrogen purging, a small amount of ascorbic acid was injected, and the mixture was stirred at 40°C for 18 h. The solvent was removed by rotary evaporation, and the residue was dissolved in methanol and added dropwise to ether. After centrifugation at 8000 rpm and 4°C for 10 minutes, the supernatant was discarded, and the precipitate was redissolved in methanol and further purified using a size exclusion chromatography. The structure of SN38-PEG-Mal with a maleimide functional group was confirmed by ^{1}H NMR spectrum (Figure S13).

Preparation of recombinant protein-drug conjugates (RPDCs)

SN38-PEG-Mal and recombinant proteins with a molar ratio of 1.2:1 was mixed in PBS at a pH of 7.4. An equimolar quantity of TCEP and EDTA was added, and the entire reaction was gently stirred at 4°C overnight. Ultrafiltration tubes with a cutoff molecular weight of 10,000 Da were first used to remove molecules with low molecular weights. Further, the crude products were purified using a dextran gel column (G-25). The SDS-PAGE demonstrate the successful synthesis of all RPs and its conjugation to the drug (Figure S14). The drug loading content was calculated based on the absorbance values at 365 nm, which is a characteristic absorption peak of SN-38, using a UV-Vis spectrophotometer (Figure S18).

The protein concentration in RPDC was derived from the following formula:

$$C_{RP} = \frac{A_{RPDC280} - A_{D280}}{b \times \varepsilon_{RP280}}$$

where b stands for the path length of the cuvette, $\varepsilon_{sRP\text{-}EHI280}$=69790 M^{-1}cm^{-1}, $\varepsilon_{lRP\text{-}HEI280}$=62340 M^{-1}cm^{-1}, $\varepsilon_{lRP\text{-}EHI280}$=62340 M^{-1}cm^{-1}. The value of A_{D280} was calculated by

the UV standard curve obtained using different concentrations of SN38-PEG-Mal.

$A_{D280} = 0.6623 \times A_{RPDC365}$

The SN38 concentration in RPDC was calculated using the following formula:

$C_D = 1.603 \times 10^{-3} \times A_{RPDC365}$

The drug loading concentration was calculated using the following formula:

$$\mathrm{LC}\,(\%) = \frac{\mathrm{M}_D C_D}{\mathrm{M}_{D'} C_{D'} + \mathrm{M}_{\mathrm{RP}} C_{RP}} \times 100\%$$

where M stands for molar mass, *C* stands for molar concentration, D stands for SN38 and D’ stands for SN38-PEG-Mal.

The molecular weights of RPDCs were confirmed by Q-TOF MS (Figure S15, S16, S17). As the bifunctional PEG linking the drugs has a relatively wide molecular weight range, the MS spectra display multiple molecular weights. It can be inferred that the synthesis of RPDCs was successful based on the molecular weight with an intensity close to 100% is close to the theoretical molecular weights corresponding to different drug-to-antibody ratio (DAR) values.

Receptor-binding affinity

The affinities of RPs binding to EGFR and HER2 were determined through biolayer interferometry (BLI) assay using the Octet® BLI system (Sartorius, Göttingen, Germany) in combination with anti-hIgG or anti-mIgG Fc Capture surface biosensors. Biosensors were loaded by exposing the tips (pre-hydrated in PBST) to samples containing receptor proteins (50 µg/mL Human EGFR with hFc-tag or HER2 with mFc-tag) for 180 s until reaching a signal threshold of 0.7 nm. Baselines were recorded for 60 s from buffer in both, drop- and tube position. Association of samples containing increasing amounts RPs to loaded biosensor tips was recorded for 180 s from drop-holder position. Dissociation was measured by dipping the biosensor tip into a tube filled with buffer for 180 s. Reference measurements were conducted by using buffer instead of RPs samples. The sample-sensorgrams were corrected by subtracting the reference curve. Global 1:1 fitting of association- and dissociation curves with software

revealed K_D. At least five different concentrations of each RP sample have been measured and globally fitted for accurate dissociation constant (K_D) determinations of the interactions between RPs and EGFR or HER2.

The affinities of RPs binding to integrin $\alpha_v\beta_3$ were measured through isothermal titration calorimetry (ITC) assay using a Malvern MicroCal ITC200-09-11-547 instrument at 25 ºC. Integrin $\alpha_v\beta_3$ (final concentrations: 0.2 μM) and RPs (final concentrations: 20 μM) were first dissolved in 1× PBS (pH 7.4). Then, the solution of RPs and integrin was filtered using sterile filters with a pore size of 0.22 μm. Approximately 300 μL RPs solution was loaded in the cell of the ITC instrument, and 40 μL integrin $\alpha_v\beta_3$ solution was loaded in the titration syringe. After a 60s pre-delay period, a 0.4 μL purge injection was performed and followed up with nineteen 2 μL injections of cofactor solution. Interval between each injection was set to 150s and stirring speed was set to 1000 rpm. The data recorded by the instrument was analyzed using the accompanying malvern analysis software and fit with a one-site binding model.

Cell Internalization

Cells were seeded in 6-well plates at a density of 1×10^5 cells per well with one coverslip placed in each well. The plates were then cultured overnight in a humid environment at 37°C with 5% CO_2. Subsequently, 200 μL rhodamine B labeled recombinant proteins with equal fluorescence intensities were added to each well and incubated at 37°C with 5% CO_2 for 24 hours. After removing the culture medium, the cells were rinsed three times with PBS and fixed with 4% paraformaldehyde at room temperature for 8-10 minutes. Following three additional PBS washes, the cells were stained with DAPI for 8-10 minutes at room temperature. After rinsing with PBS, the coverslips were placed in PBS. The cells were then observed under a confocal laser scanning microscope (LSM-710).

For lysosome staining, 200 nM Lyso-Tracker Green was added into cells for another 1 h, followed with 24 h incubation with rhodamine B labeled RPDCs. Then, the cells

were washed, fixed and observed using a CLSM.

Flow Cytometry

The human tumor cell lines including MCF-7, HeLa, and A549, were seeded at a density of 5×10^4 cells per well in 24-well plates and incubated with RPs for 4 h at 37°C in a humidified environment with 5% CO_2. The culture supernatant was discarded, and the cells were washed with PBS. Subsequently, 100 µL trypsin was added to each well for 1 minute at room temperature, followed by the addition of 1 mL of culture medium to stop the digestion. The cells were gently pipetted, transferred to 1.5 mL centrifuge tubes, and centrifuged at 1000 rpm for 5 minutes, after which the supernatant was discarded. Each tube was resuspended in 1 mL of PBS and centrifuged again at 1000 rpm for 5 minutes; this step was repeated twice. The cells were then resuspended in 50 µL of prepared antibody staining solution (PE Mouse Anti-Human EGF Receptor; BV421 Mouse Anti-Human CD340 (HER-2), 1:1000; FITC Mouse Anti-Human CD51/CD61 (23C6), 1:200) and stained at room temperature for 30 minutes. After staining, the cells were centrifuged at 1000 rpm for 5 minutes and the supernatant was discarded. Finally, the cells were resuspended in 150 µL of PBS. For the negative control group (NC), the cells were not stained but instead resuspended in 150 µL of PBS. After filtration through a cell strainer, the samples were analyzed using a flow cytometer.

Cellular uptake pathways of RPs were determined by flow cytometry. Specifically, the cells were digested into single cell suspension and pre-co-incubated with cytochalasin D (Cyto-D, 5 µM), chlorpromazine (CPZ, 10 µM), sodium azide (NaN_3) and m-β-CD (methyl-β-cyclodextrin) (M-β-CD, 300 µM), respectively, for 1 hour. Subsequently, FITC labeled RPs were added to the cells for another 1 hour. After filtration through a cell strainer, the samples were analyzed by a flow cytometer.

Cell Viability

Cells were seeded at a density of 5,000 cells per well in a 96-well plate and cultured

overnight at 37°C in a humidified atmosphere with 5% CO_2. Subsequently, the cell culture medium was replaced with medium containing various concentrations of drugs. Wells with cells but without drugs were used as control. After 48 hours of incubation, the culture medium was removed, and fresh medium was added, along with 20 μL of MTT solution (5 mg/mL in PBS). The cells were incubated for another 4 hours at 37°C. The liquid from each well was aspirated, and 150 μL DMSO was added. After 10 minutes, the absorbance at 490 nm for each well was measured using a microplate reader. Cell viability was calculated using the following formula:

Cell Viability (%) = A_{490} of test wells / A_{490} of control wells × 100%

Where A_{490} of test wells and A_{490} of control wells represent the values with background well absorbance subtracted.

Western Blotting

Cells were evenly seeded at a density of 1×10^5 cells per well in six-well plates and cultured in DMEM medium containing 10% fetal bovine serum overnight at 37°C in a humidified environment with 5% CO_2. The culture medium was then replaced, and 200 μL of each sample was added. PBS was added as a negative control group. The six-well plates with the added samples were continued to be cultured for 24 hours at 37°C in a humidified environment with 5% CO_2, followed by protein extraction. The protein extraction method involved removing the culture medium mixture from the plates, washing three times with pre-cooled PBS, adding cell lysis buffer for light-protected disruption, mixing the lysate by pipetting, and centrifuging at 4°C.

20 μL of each sample was loaded per lane, and electrophoresis was conducted at a concentration gel voltage of 100 V for 30 minutes, followed by separation gel electrophoresis at an increased voltage of 130 V for one hour. After electrophoresis, the SDS-PAGE gel was peeled off and subjected to wet transfer. The transfer solution was pre-cooled to 4°C, and the transfer was performed at a constant current of 200 mA for 90 minutes. After completion, the membrane was taken out and washed in TBST solution for 5 minutes, followed by incubation in blocking solution at room temperature

for 1 hour. After blocking, incubation with the primary antibody overnight and secondary antibody for one hour was performed separately. The membrane was then washed three times with TBST solution, co-incubated with the developing solution for 5 minutes, and exposed and photographed using the instrument.

In Vivo Anti-Tumor Experiments

20-25 g female nude mice were used for the anti-tumor experiments. To establish a subcutaneous HeLa tumor model, 1×10^7 HeLa cells in 100 μL of physiological saline were subcutaneously injected into mice. Approximately three and five weeks after injection, when the tumors reached a size of approximately 50-100 mm^3 or over 100 mm^3. The tumor models were successfully established and the tumor-bearing mice were randomly divided into several groups, each consisting of 3 mice, with no significant differences in body weight.

For small tumor model, sPRDC-EHI, lRPDC-EHI, and lRPDC-HEI with equal SN-38 concentration were injected into the tumor-bearing mice via the tail vein at a dose of 0.4 mg/kg (calculated based on SN-38). And the mice treated with PBS were used as controls. All agents were administered every two days for a total of six dose. For large tumor model, sPRDC-EHI and lRPDC-HEI with equal SN-38 concentration were injected into the tumor-bearing mice via the tail vein at a dose of 0.8 mg/kg (calculated based on SN-38). And the mice treated with PBS were used as controls. All agents were administered every two days for a total of four dose.

Tumor diameter was measured every two days using calipers, and tumor volume (V) was calculated using the formula: $V = (ab^2) / 2$, where 'a' and 'b' corresponded to the longest and shortest diameters of the tumor, respectively. Mouse body weights were recorded until the end of the anti-tumor experiment.

NIR imaging and *In Vivo* Distribution Experiment

A subcutaneous HeLa tumor model was established as above. To visualize the biodistribution of sRPDC-EHI *in vivo*, sRPDC-EHI was first labeled with a near-

infrared fluorescent probe, NIR-797. Subsequently, a 500 μL solution of NIR-797-labeled sRPDC-EHI was administered via tail vein injection into the mice. At designated time points, the mice were anesthetized, and real-time near-infrared imaging was performed using the IVIS imaging system. At the end of the experiment, the mice's organs were harvested, and imaging was conducted using the IVIS imaging system.

Pharmacokinetic Analysis *In Vivo*

The pharmacokinetic profile of sRPDC-EHI was evaluated in male ICR mice following intravenous administration. Cy5.5-labeled sRPDC-EHI was administered via tail vein injection at a dosage of 0.4 mg/kg (calculated based on SN38). Blood samples were collected using heparin sodium-coated collection tubes at designated time points (5 min, 10 min, 30 min, 1 h, 2 h, 4 h, 8 h, and 24 h post-injection). The blood samples were then centrifuged at 3,500 rpm for 15 minutes to collect serum. For quantitative analysis, 200 μL of serum from each time point was mixed with an extraction solvent composed of methanol and water (methanol: water= 7:3) and diluted to a final volume of 5 mL. The mixture was incubated at room temperature for 24 hours to facilitate analyte extraction and the supernatant was collected after subsequent centrifugation, and the concentration of sRPDC-EHI-Cy5.5 was determined via fluorescence spectrophotometry.

Toxicity Studies and Immunogenicity Assessment *In Vivo*

sPRDC-EHI, lRPDC-EHI, and lRPDC-HEI were injected into the ICR mice via the tail vein at a dose of 0.4 mg/kg (calculated based on SN-38). And the mice treated with PBS were used as controls. All agents were administered every two days for a total of six dose. Blood samples were collected at day7 post final injection. The complete blood count (CBC) analysis including red blood cells (RBC), white blood cells (WBC), red blood cell distribution width (RDW), mean corpuscular hemoglobin concentration (MCHC), mean corpuscular hemoglobin (MCH), hemoglobin (HGB), hematocrit (HCT), mean corpuscular volume (MCV), lymphocyte count (Lymph #), granulocyte

count (Gran #), monocyte percentage (Mon%), granulocyte percentage (Gran%) and serum biochemistry indexes including aminotransferase (ALT), aspartate aminotransferase (AST), blood urea nitrogen (BUN), and creatinine (CREA) were analyzed. The levels of serum inflammatory cytokines including TNF-α, IL-1β and IL-6 were analyzed through ELISA. Moreover, the major organs (heart, liver, spleen, kidney, and lung) were dissected, cleaned and then stained with hematoxylin and eosin (H&E).

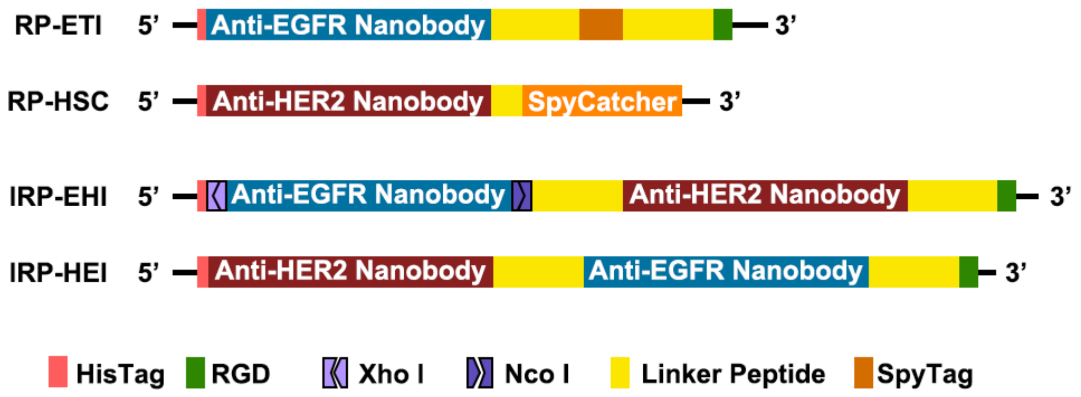

Figure S1. The plasmids of RP-ETI, RP-HSC, lRP-HEI and lRP-EHI.

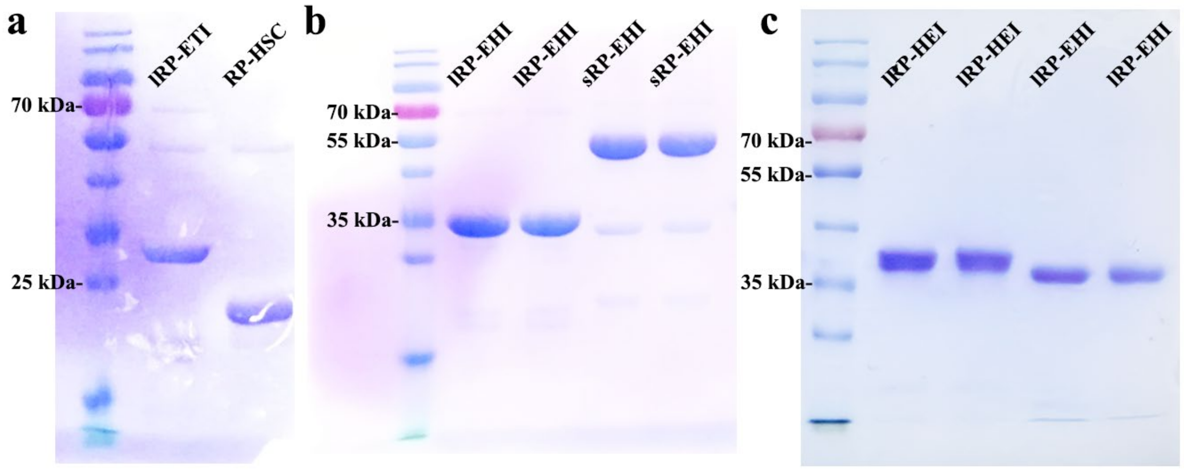

Figure S2. SDS-PAGE of RP-HSC and RP-ETI (a), lRP-EHI and sRP-EHI (b), and lRP-HEI (c).

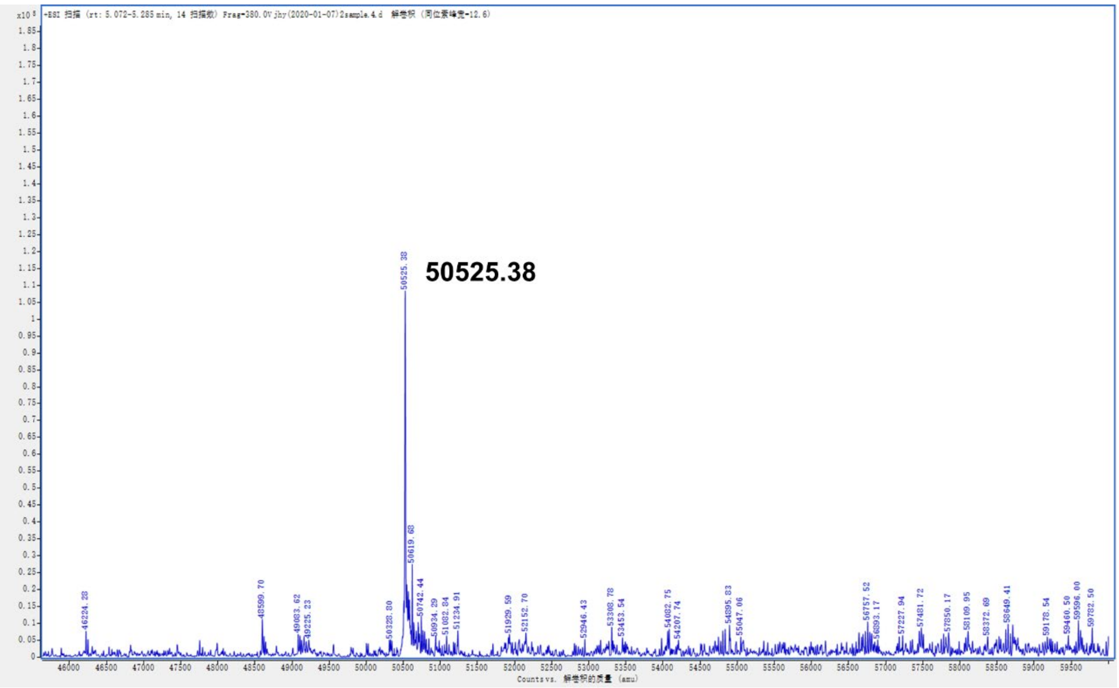

Figure S3. Q-TOF MS of sRP-EHI.

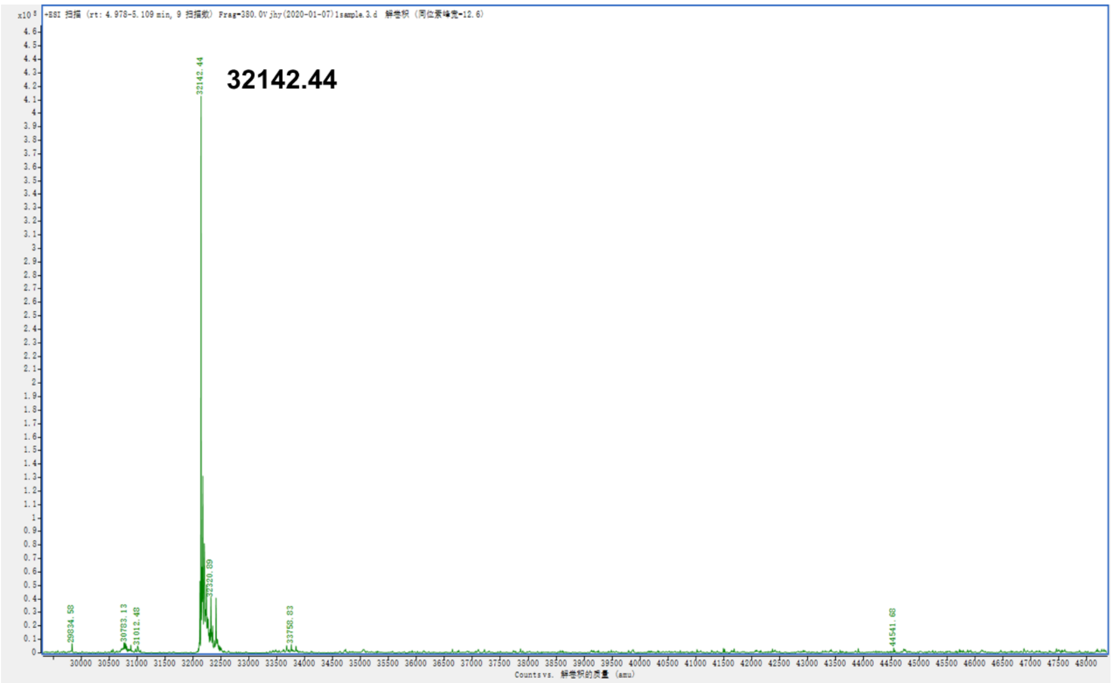

Figure S4.Q-TOF MS of lRP-EHI.

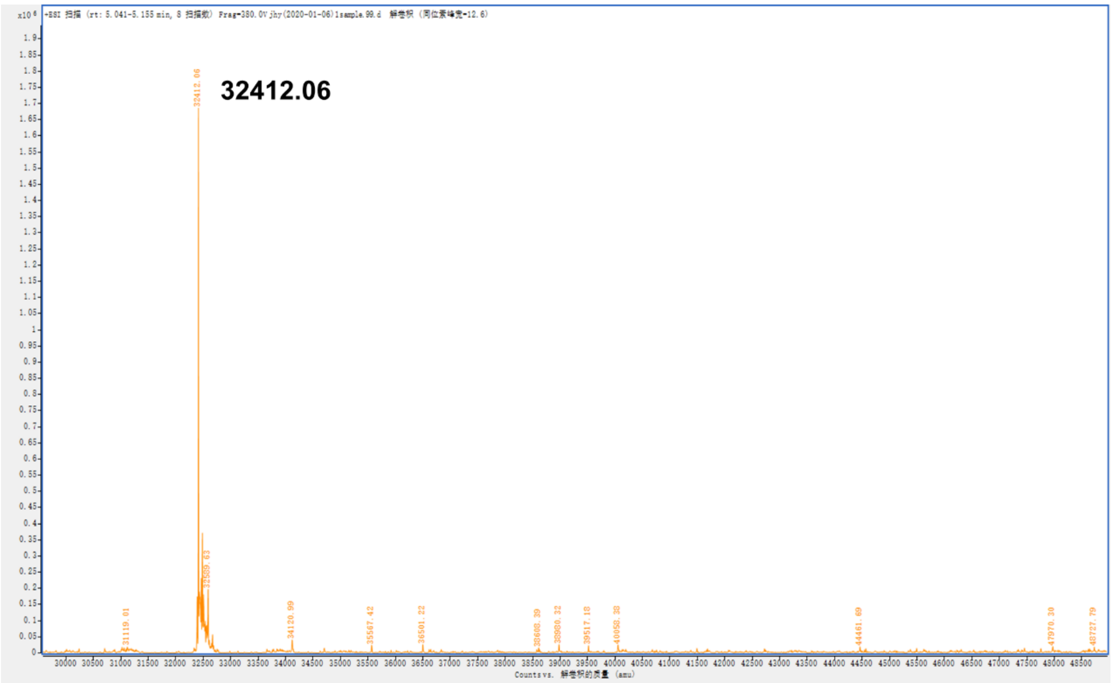

Figure S5. Q-TOF MS of lRP-HEI.

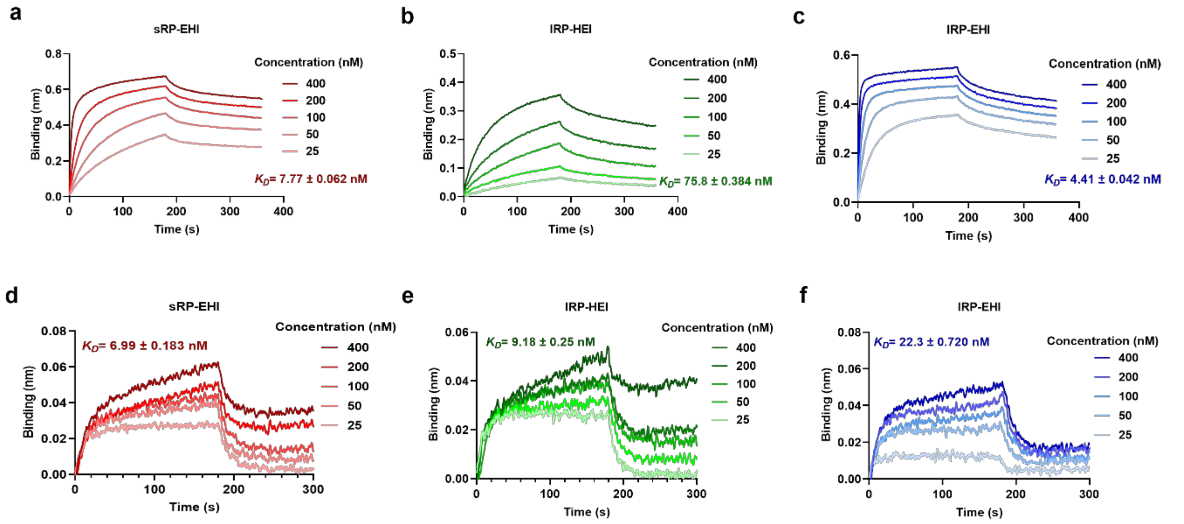

Figure S6. Biosensor dose-dependent curves of RPs binding to the immobilized EGFR and HER. 25, 50, 100, 200 and 400 nM of RPs were used. Sensorgrams show binding of RPs to the immobilized EGFR and HER2 (first 180 s) followed by the dissociation from the receptor surface. Data are presented as $K_D \pm K_D$ error.

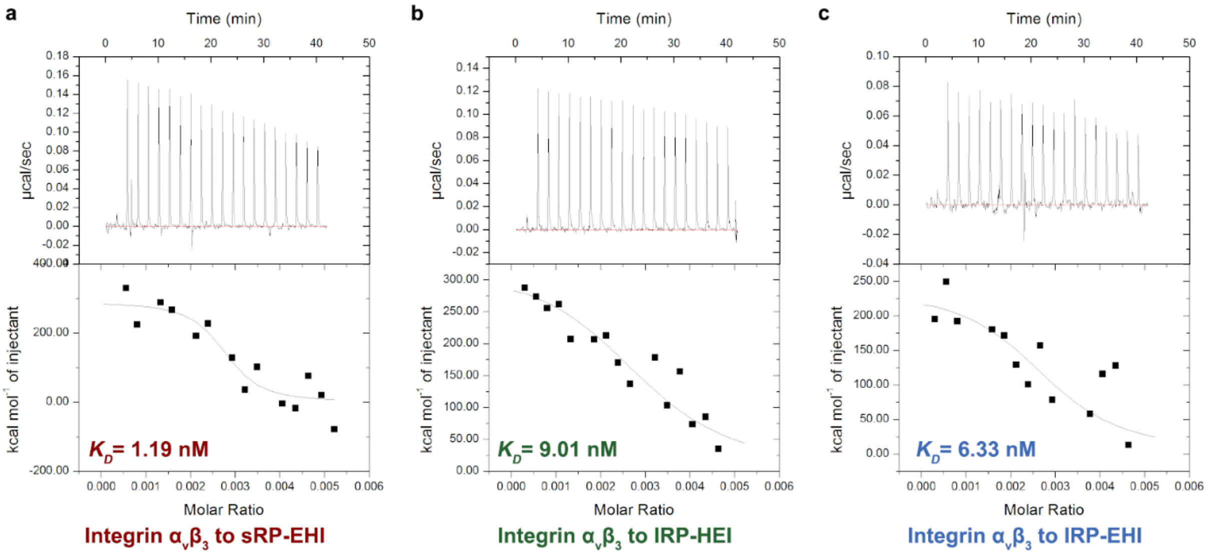

Figure S7. The raw thermogram and binding isotherm obtained in the ITC assay for analysis of the interactions between integrin $\alpha_v\beta_3$ and sRP-EHI (a), lRP-HEI (b) and lRP-EHI (c).

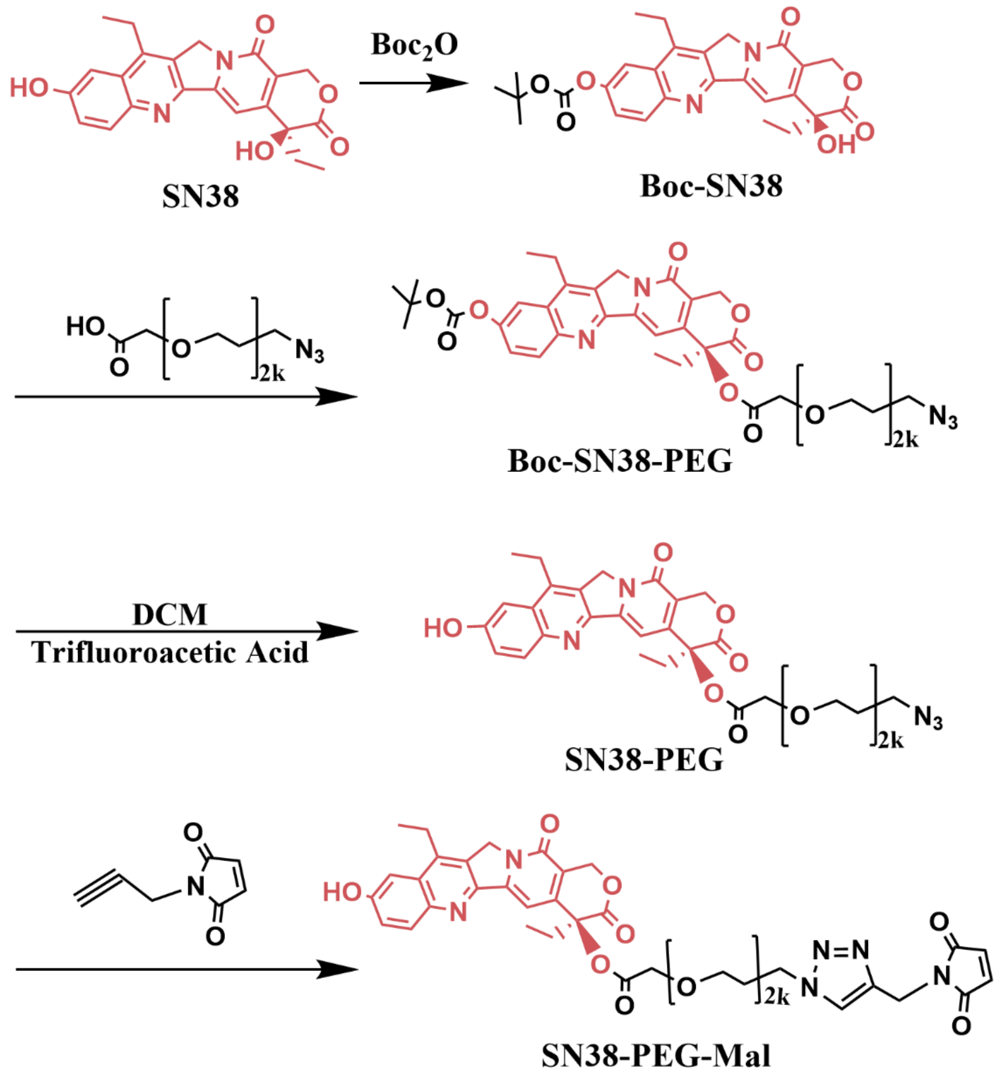

Figure S8. Synthesis route of SN38-PEG-Mal.

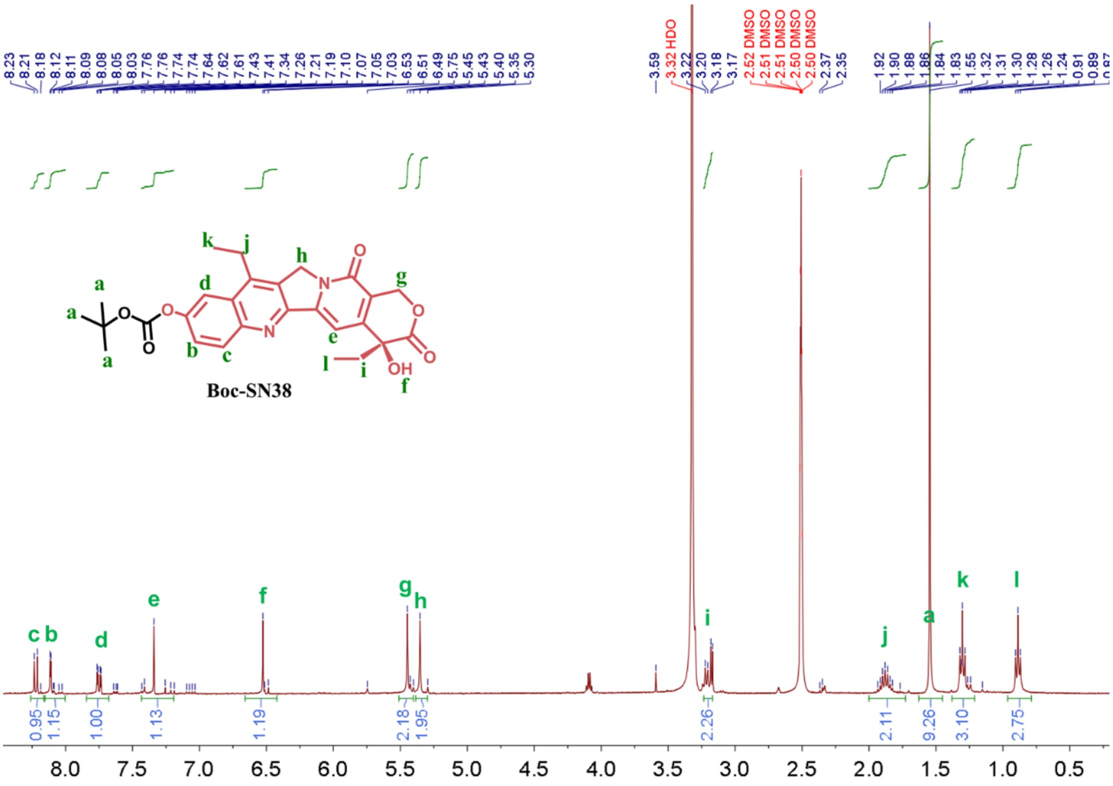

Figure S9. ^{1}H NMR Spectrum of Boc-SN38 (in DMSO-d_6).

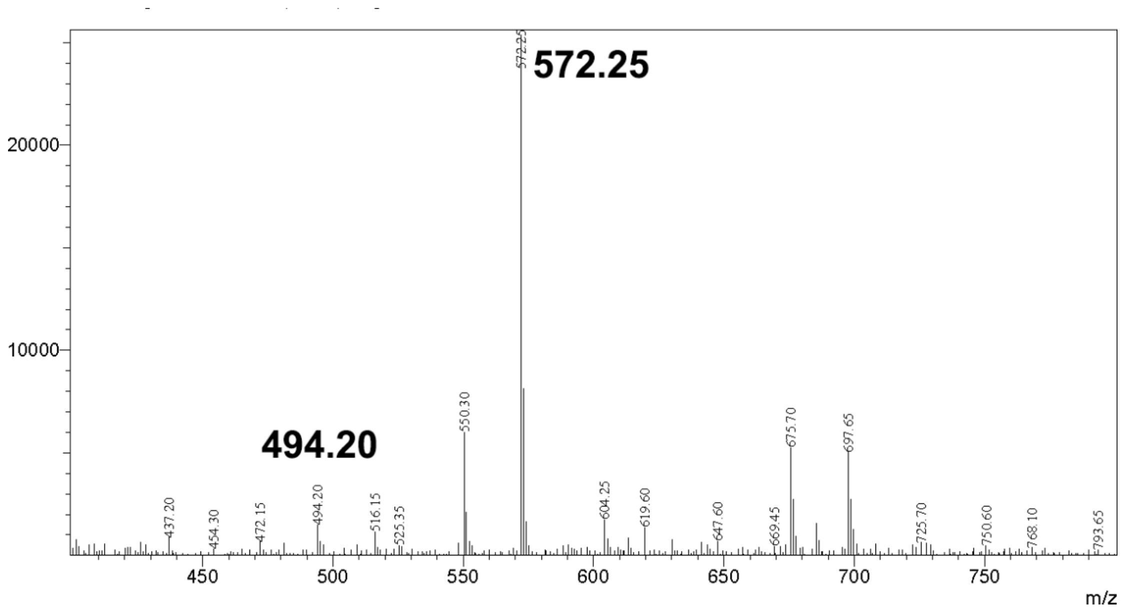

Figure S10. Electrospray lonization mass spectrum of Boc-SN38.

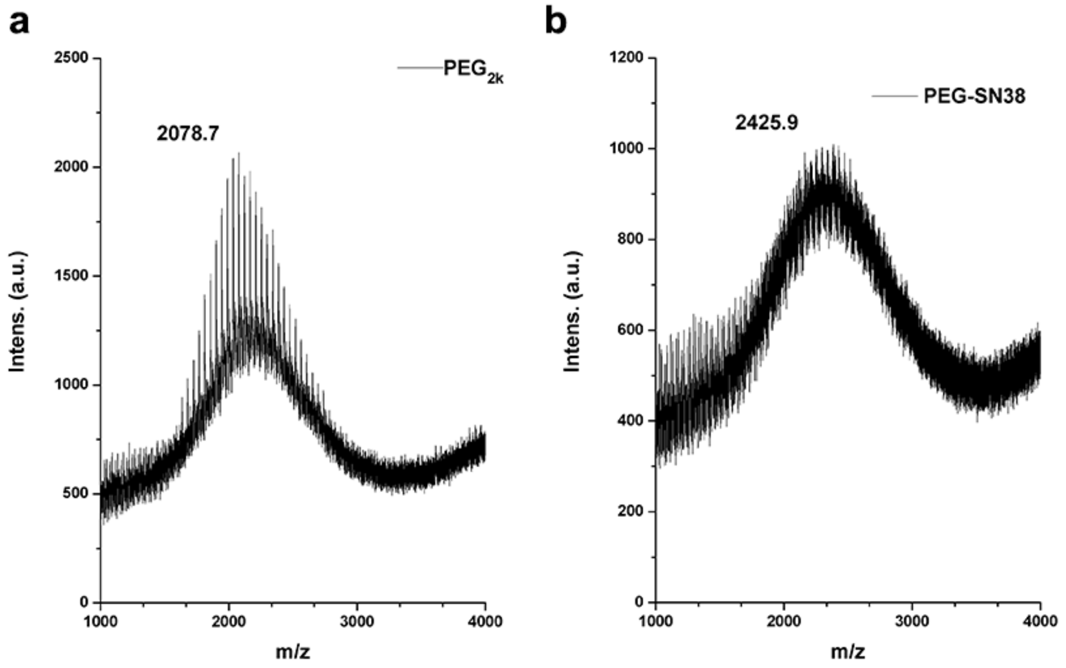

Figure S11. MALDI-TOF of PEG_{2k} and SN38-PEG. Measured at 50% acetonitrile aqueous solution containing 0.1 % trifluoroacetic acid, and sinapic acid was used as the matrix.

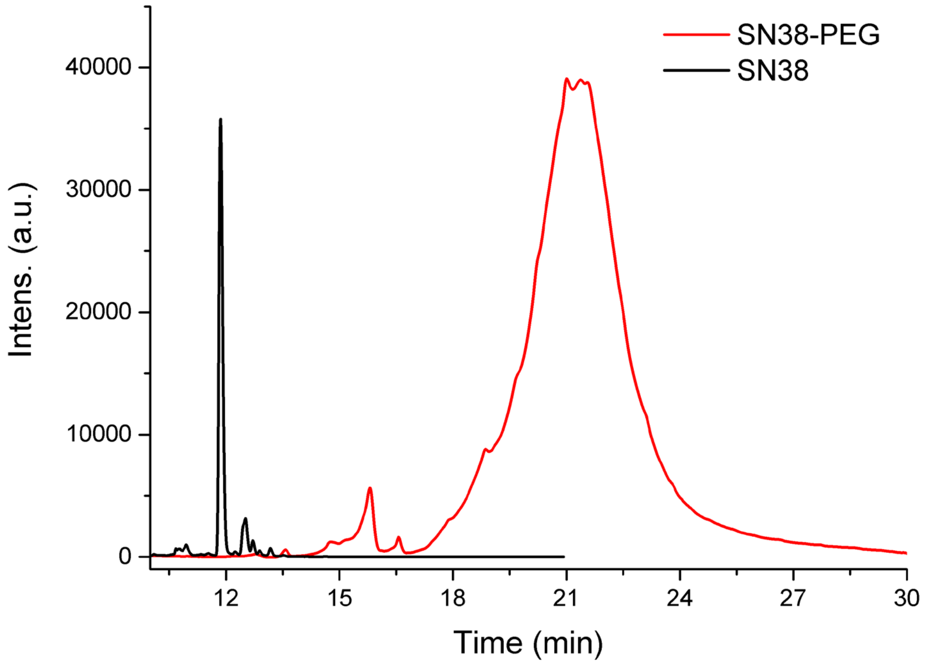

Figure S12. HPLC of SN38 and SN38-PEG.

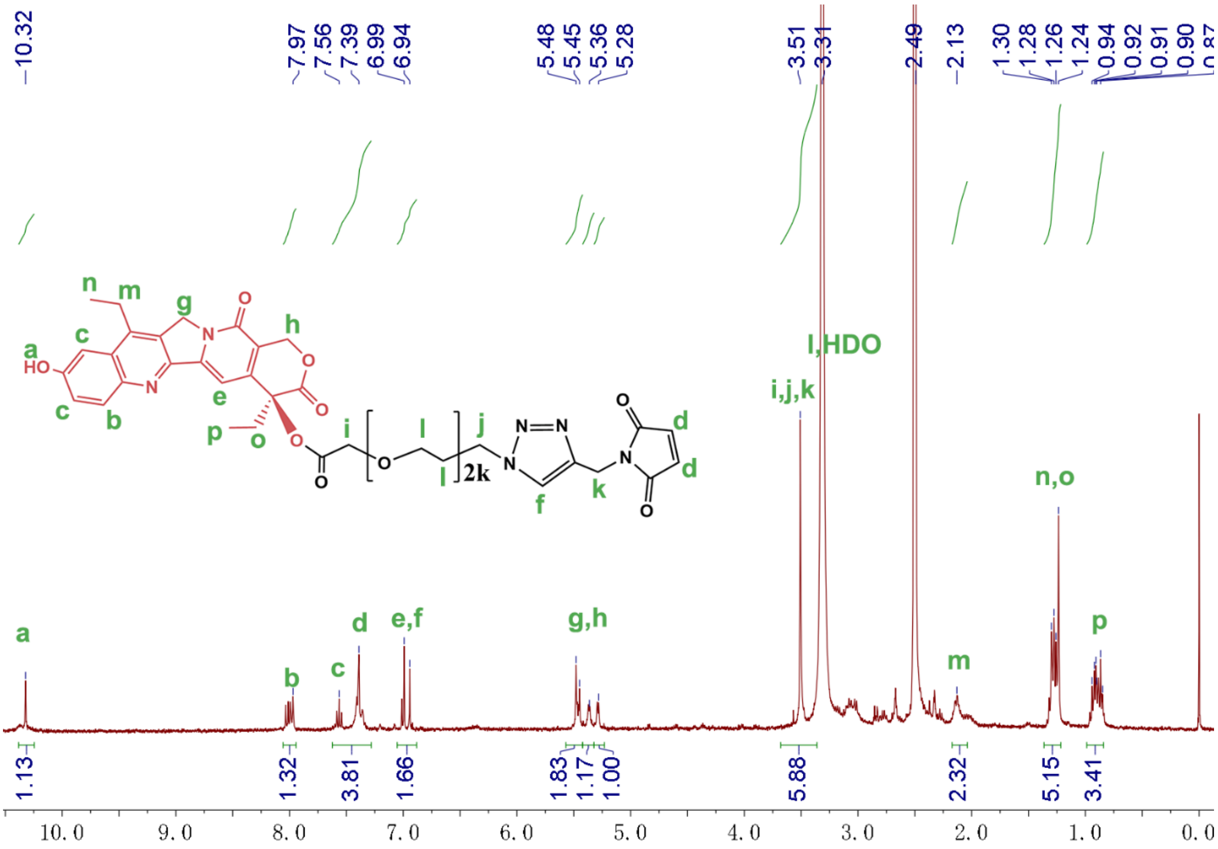

Figure S13. ^{1}H NMR spectrum of SN38-PEG-Mal (in DMSO-d_6).

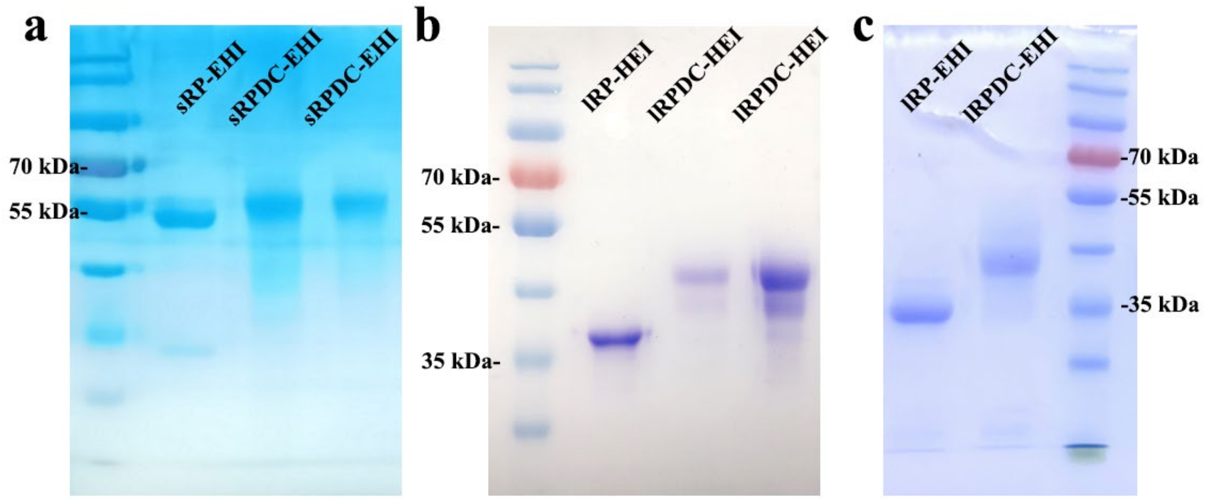

Figure S14. SDS-PAGE of sRP-EHI and sRPDC-EHI (a), lRP-HEI and lRPDC-HEI (b), lRP-EHI and lRPDC-EHI (c).

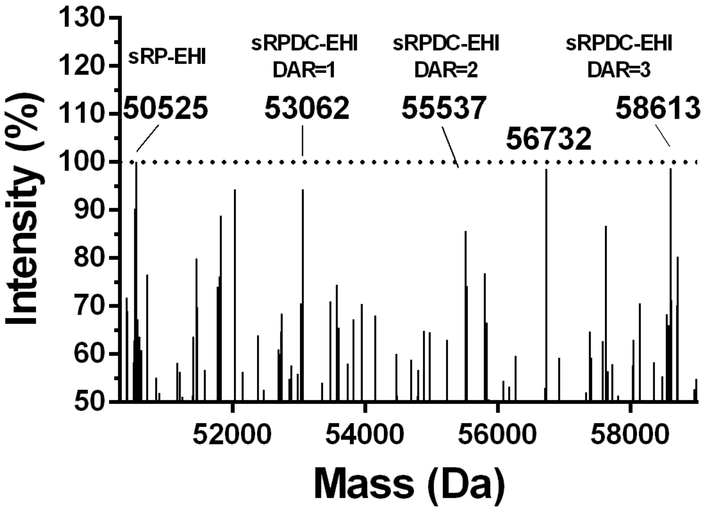

Figure S15. Q-TOF MS of sRPDC-EHI.

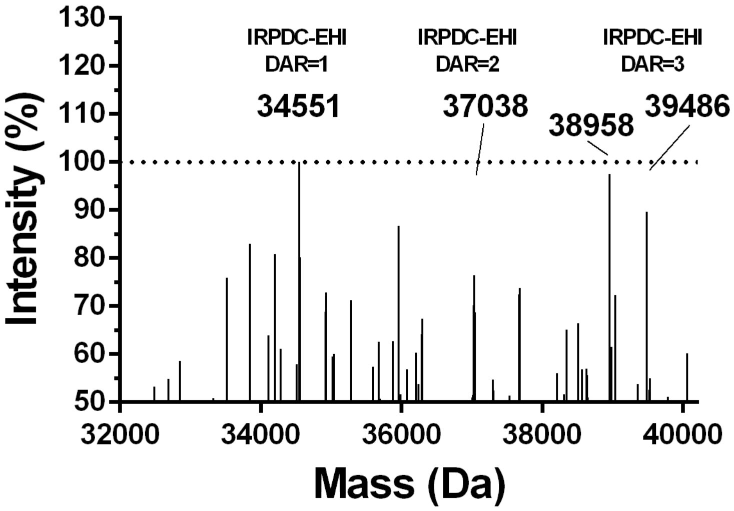

Figure S16. Q-TOF MS of lRPDC-EHI.

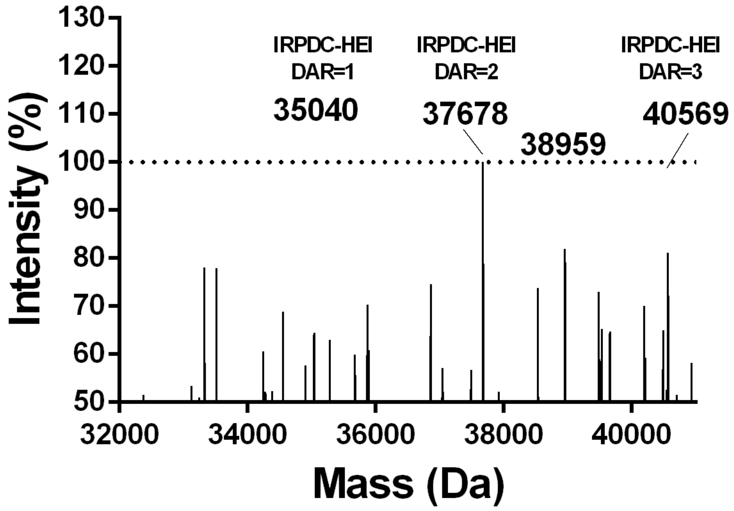

Figure S17. Q-TOF MS of lRPDC-HEI.

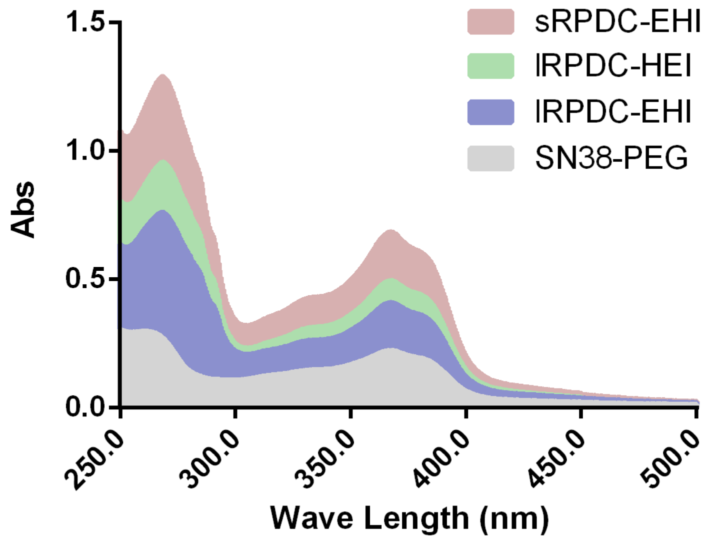

Figure S18. UV absorption spectra of sRPDC-EHI, lRPDC-EHI, lRPDC-HEI and SN38-PEG.

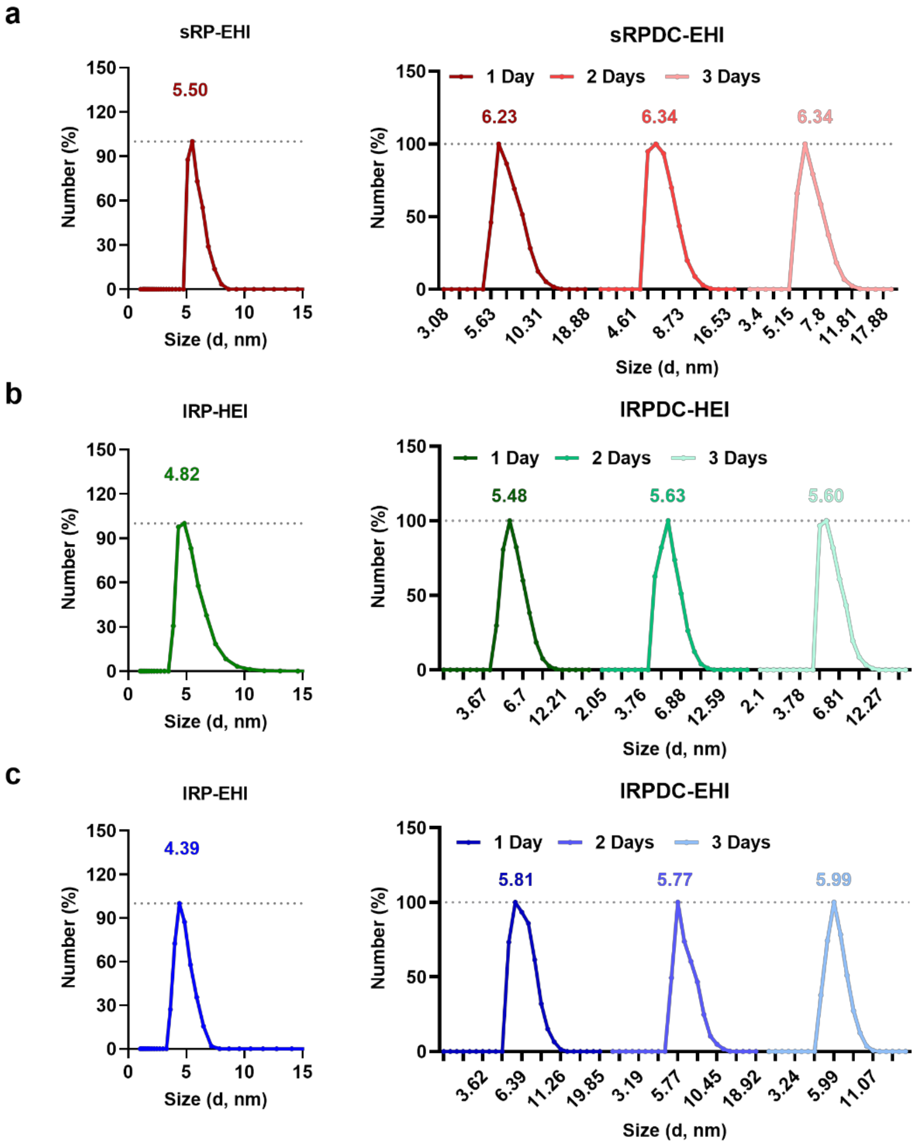

Figure S19. The dynamic light scattering (DLS) analysis of RPDCs demonstrated their stability (RT,PBS), with no observed aggregation or degradation.

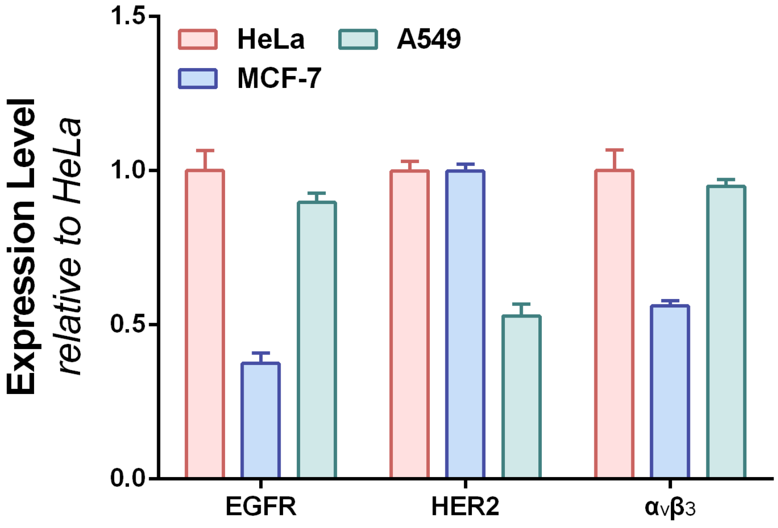

Figure S20. Biomarkers expression in A549, HeLa, and MCF-7 cells. Data are presented as mean ± SD, n = 3.

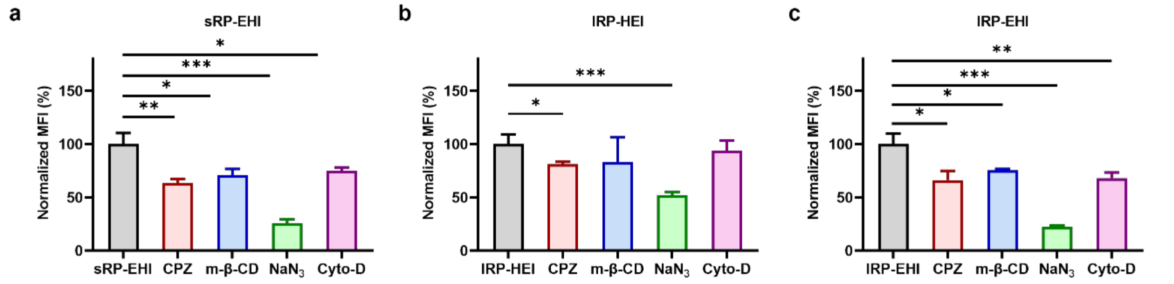

Figure S21. The endocytosis pathways of sRP-EHI (a), lRP-HEI (b) and lRP-EHI (c) in HeLa cells evaluated by a flow cytometry. Data are presented in mean ± SD, statistical significances were calculated using unpaired *t*-test, n = 3, *$p < 0.05$, **$p < 0.01$, ***$p < 0.001$, ****$p < 0.0001$.

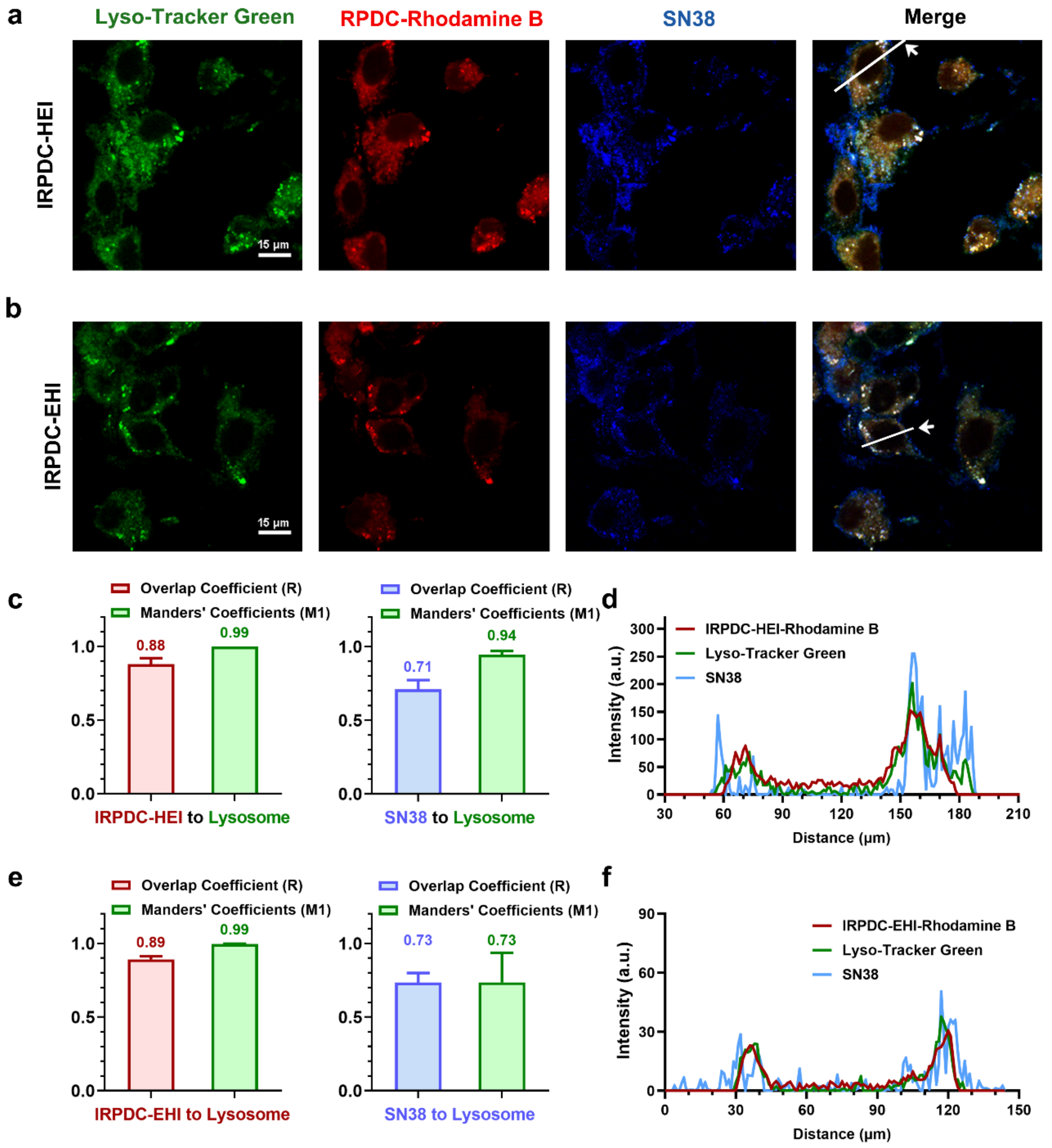

Figure S22. Lysosome colocalization study of IRPDCs in HeLa cells. (a-b) CLSM images of HeLa cells incubated with rhodamine B labeled IRPDC-HEI (red) (a), IRPDC-EHI (b) for 24 h, followed with 1 h incubation with Lyso-Tracker Green (green). SN38 (blue) was observed through its autofluorescence at 405 nm. Scale bar, 15 μm. (c) Overlap coefficient (R) and Manders' coefficients of the images in (b). (e) Overlap coefficient (R) and Manders' coefficients of the images in (b). (f) Fluorescence intensity profiles along the designated lines in (b). Data are presented in mean ± SD. n=4.

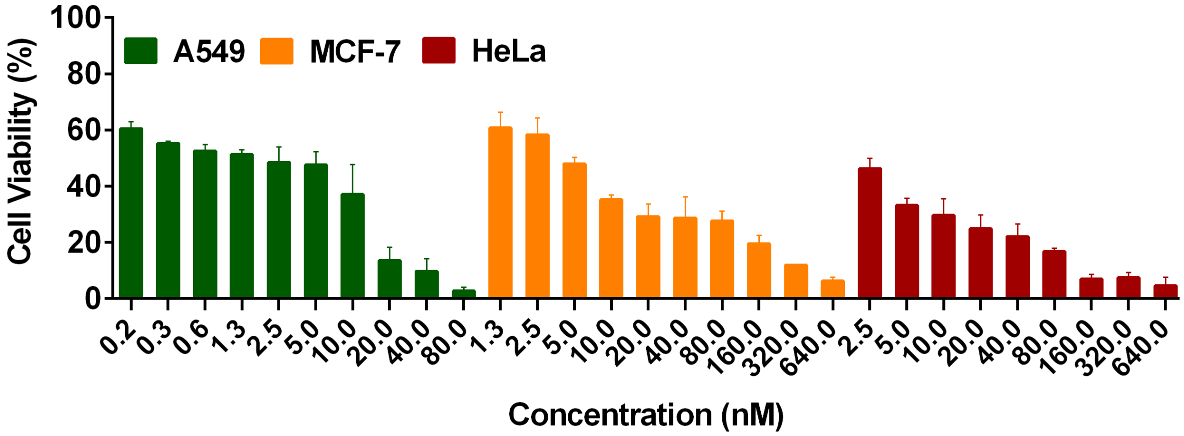

Figure S23. *In vitro* cytotoxicity of SN38 against A549 (left), MCF-7 (middle) and HeLa (right) cells with various concentration after incubation for 48 h. Data are presented as mean ± SD, n = 3, statistical significances were calculated using multiple *t*-tests, $**p < 0.01$, $***p < 0.001$, $****p < 0.0001$.

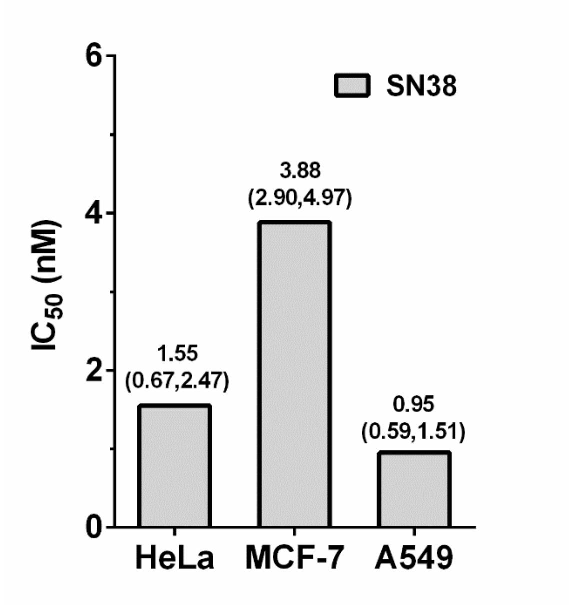

Figure S24. The IC_{50} values of SN38 against different cancer cells after 48 h incubation. Data are presented as IC_{50} (95% Confidence Intervals), n≥3.

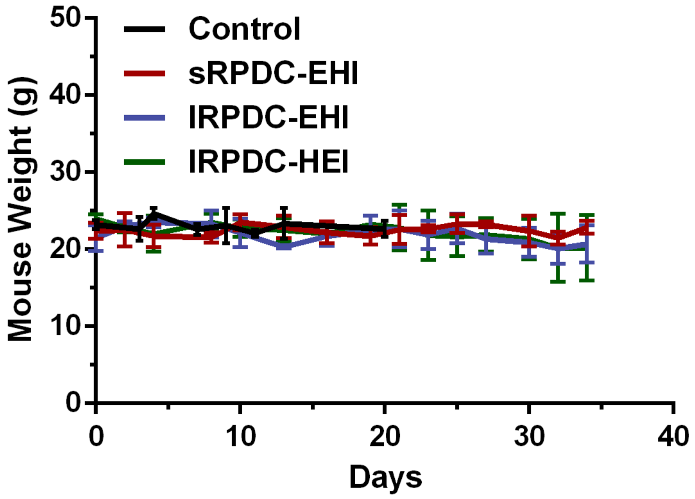

Figure S25. Body weight of mice bearing small HeLa tumors treated with different agents. Data are presented as mean ± SD. n =3.

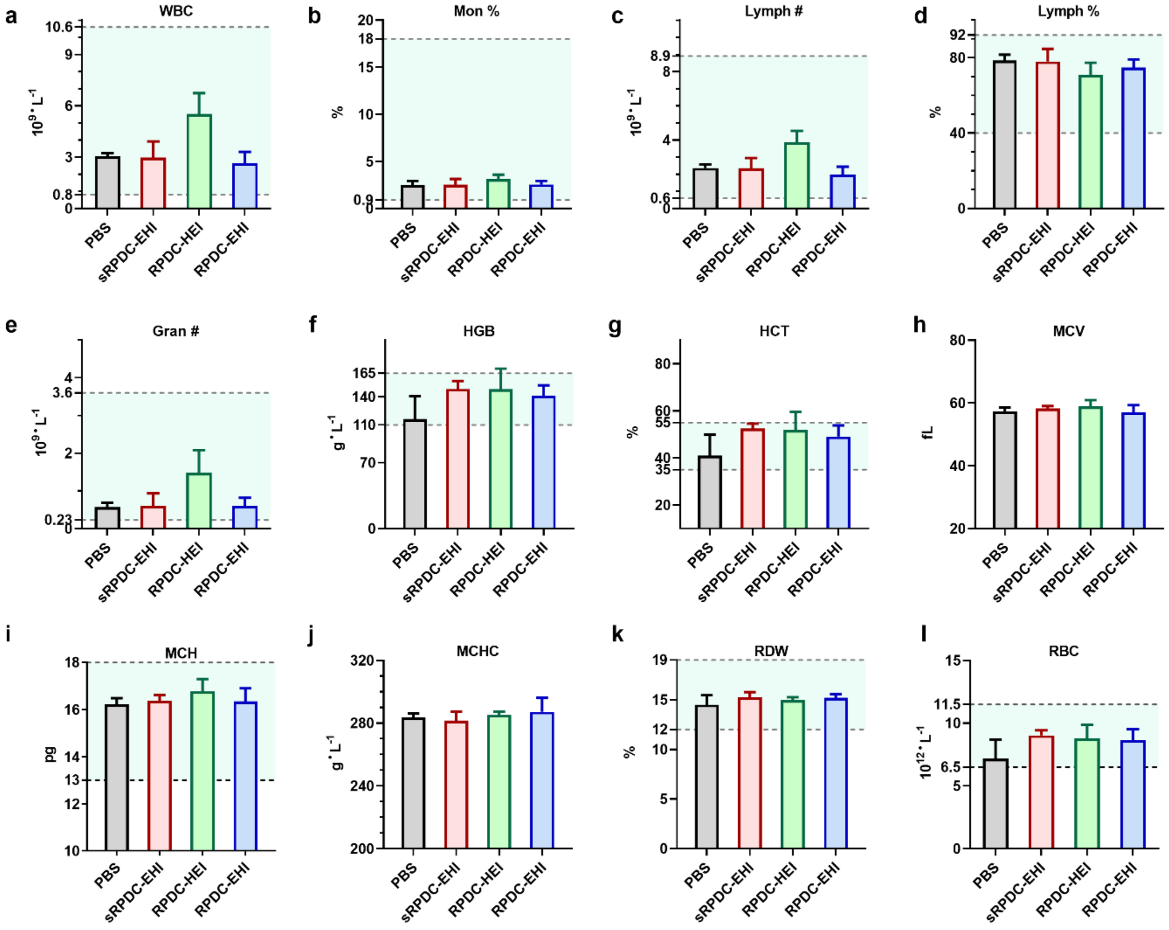

Figure S26. (a-l) Comprehensive analysis of complete blood count of RPDCs-treated mice. Data are presented as mean ± SD, n = 3.

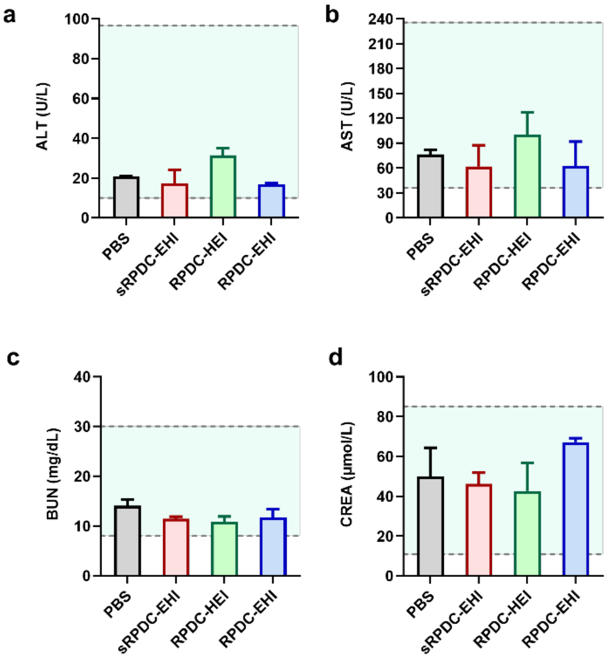

Figure S27. (a-d) Blood biochemistry of RPDCs-treated mice. Data are presented as mean ± SD, n = 3.

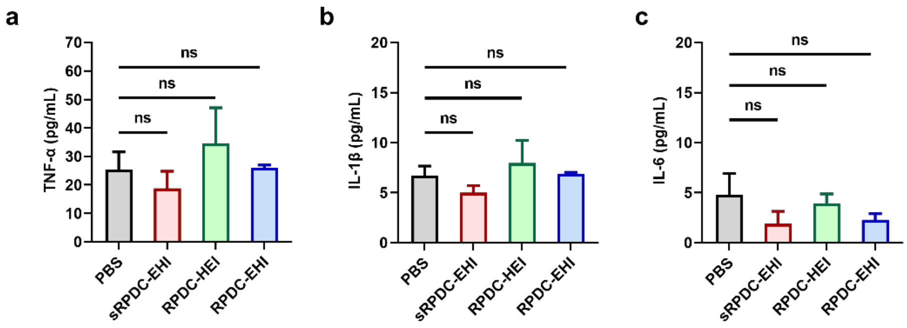

Figure S28. The levels of serum inflammatory cytokines of RPDCs-treated mice. The levels of TNF-α (a), IL-1β (b), and IL-6 (c) of the RPDCs treated mice for 7 days were examined by ELISA. Data are presented as mean ± SD, n = 3. Statistical significance was calculated by *t*-test. n.s., not significant.

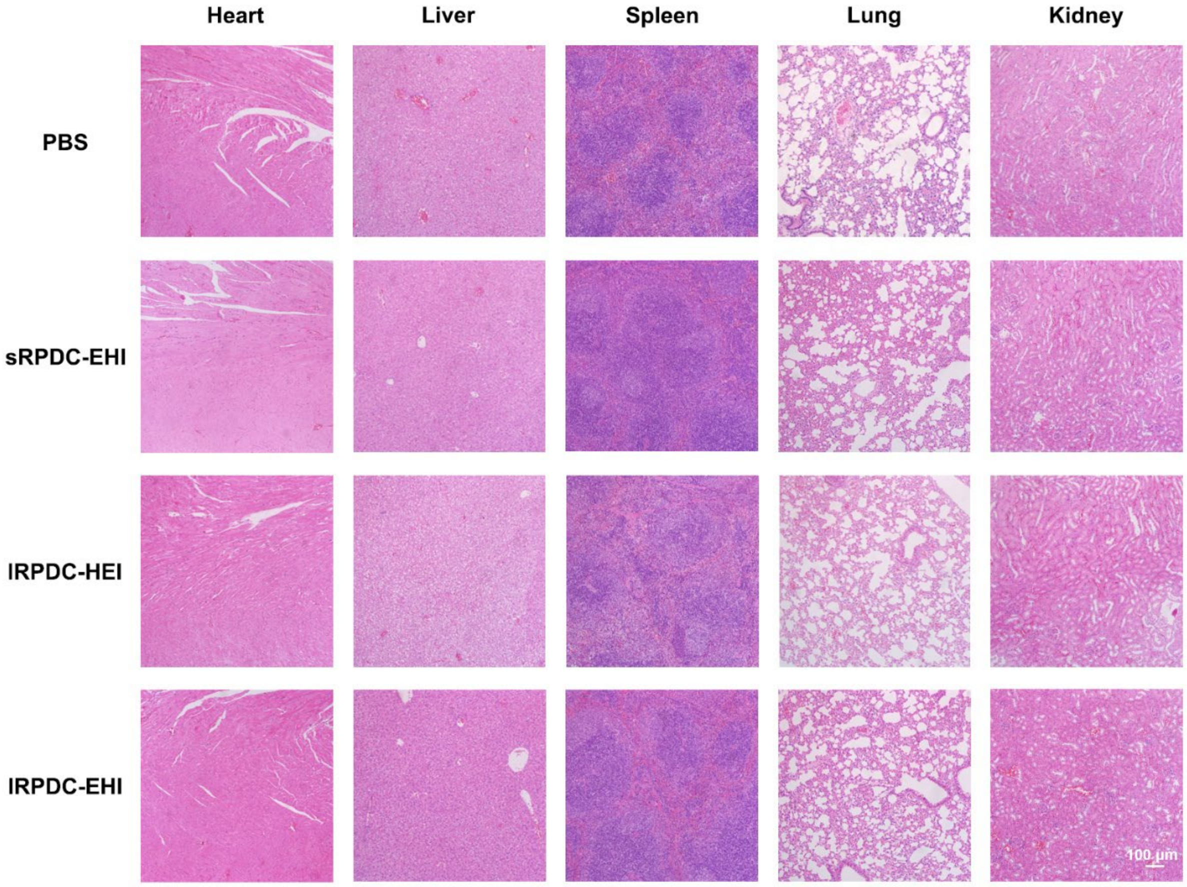

Figure S29. H&E staining of RPDCs-treated mouse tissues.

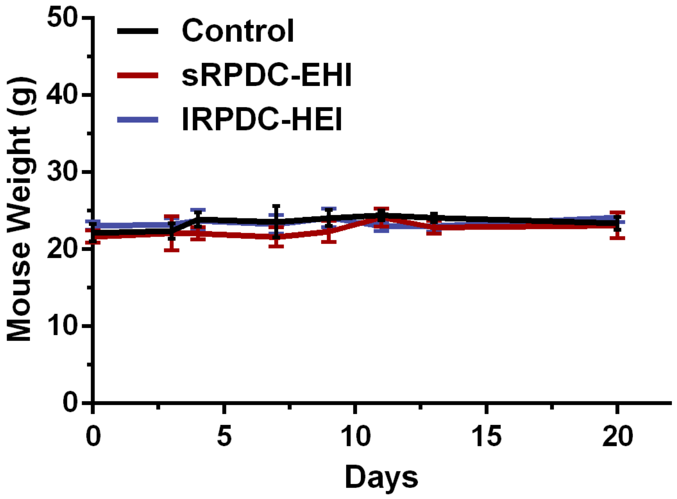

Figure S30. Body weight of mice bearing large HeLa tumors treated with different agents. Data are presented as mean ± SD. n =3.

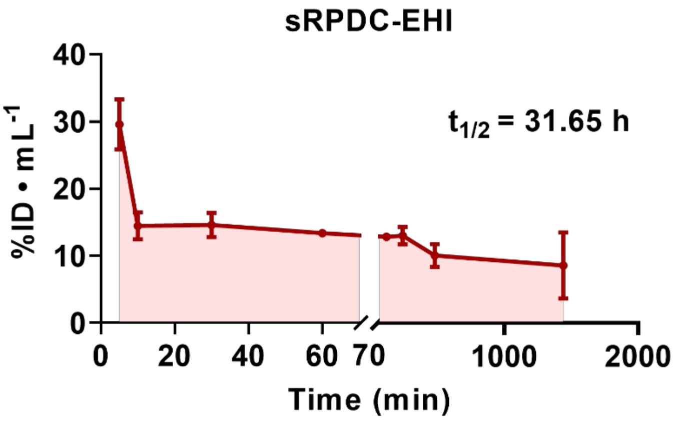

Figure S31. Pharmacokinetic analysis of sRPDC-EHI *in vivo*. Data are presented as mean ± SD, n = 3.

References

[1] C. H. Whang, E. Yoo, S. K. Hur, K. S. Kim, D. Kim, S. Jo, *Chem. Commun.* **2018**, *54*, 9031-9034.
[2] X. Liu, J. Wang, Y. Shen, J. Tang, M. Sui, *J. Control. Release* **2015**, *213*, e124.
